# An Otx/Nodal Regulatory Signature for Posterior Neural Development in Ascidians

**DOI:** 10.1371/journal.pgen.1004548

**Published:** 2014-08-14

**Authors:** Agnès Roure, Patrick Lemaire, Sébastien Darras

**Affiliations:** 1 Institut de Biologie du Développement de Marseille, Aix-Marseille Université, CNRS UMR 7288, Campus Luminy, Marseille, France; 2 Sorbonne Universités, UPMC Univ Paris 06, UMR 7232, BIOM, Observatoire Océanologique, Banyuls/Mer, France; 3 CNRS, UMR 7232, BIOM, Observatoire Océanologique, Banyuls/Mer, France; New York University, United States of America

## Abstract

In chordates, neural induction is the first step of a complex developmental process through which ectodermal cells acquire a neural identity. In ascidians, FGF-mediated neural induction occurs at the 32-cell stage in two blastomere pairs, precursors respectively of anterior and posterior neural tissue. We combined molecular embryology and *cis*-regulatory analysis to unveil in the ascidian *Ciona intestinalis* the remarkably simple proximal genetic network that controls posterior neural fate acquisition downstream of FGF. We report that the combined action of two direct FGF targets, the TGFβ factor Nodal, acting via Smad- and Fox-binding sites, and the transcription factor Otx suffices to trigger ascidian posterior neural tissue formation. Moreover, we found that this strategy is conserved in the distantly related ascidian *Phallusia mammillata*, in spite of extreme sequence divergence in the *cis*-regulatory sequences involved. Our results thus highlight that the modes of gene regulatory network evolution differ with the evolutionary scale considered. Within ascidians, developmental regulatory networks are remarkably robust to genome sequence divergence. Between ascidians and vertebrates, major fate determinants, such as Otx and Nodal, can be co-opted into different networks. Comparative developmental studies in ascidians with divergent genomes will thus uncover shared ascidian strategies, and contribute to a better understanding of the diversity of developmental strategies within chordates.

## Author Summary

The Chordate phylum groups vertebrates, tunicates (including ascidians) and cephalochordates (amphioxus). These animals share a typical body plan characterized by the presence during embryonic life of a notochord and a dorsal neural tube. Ascidians, however, took a significantly different evolutionary path from other chordates resulting in divergent morphological, embryological and genomic features. Their development is fast and stereotyped with very few cells and ascidian genomes have undergone compaction and extensive rearrangements when compared to vertebrates, but also between ascidian species. This raises the question of whether developmental mechanisms controlling typical chordate structure formation are conserved between ascidians and vertebrates. Here, we have studied the set of ascidian genes which control the formation of the posterior part of the nervous system. We uncovered original usages of the signaling molecule Nodal and the transcription factor Otx. For example, Otx, which is a specific determinant of anterior identity in most metazoans, has been co-opted for the formation of the ascidian posterior nervous system. These two factors define a regulatory signature found in enhancers of posterior neural genes in two genomically divergent ascidian species.

## Introduction

Neural tissue formation is a multi-step process through which embryonic cells acquire a neural phenotype. In vertebrate central nervous system (CNS) development, the first step is called neural induction. Naive ectodermal cells undergo a binary fate decision between epidermis and neural tissue in response to endomesodermal signals that modulate the FGF, BMP and Wnt signaling pathways [1]–[3]. While there may be variations between species, BMP inhibition together with FGF signaling activation are key events in neural induction. Concomitantly or following neural induction, neural tissue is patterned along the antero-posterior and medio-lateral axes. Acquisition of a differentiated neural phenotype involves further processes such as stabilization and reinforcement of the neural fate, specification of cellular identity and progression towards final differentiation. Each of these steps is controlled by complex mechanisms involving a variety of molecular players [4]–[6].

Non-vertebrate chordates include ascidians (tunicates) and amphioxus (cephalochordates). They form prototypical tadpole-like larvae with a dorsal hollow neural tube patterned similarly to vertebrates [7], [8]. The embryological process of neural induction also takes place in these animals but our current knowledge does not provide a unified view. In amphioxus, BMP activation represses neural tissue formation but FGF inhibition does not abolish neural tissue formation [9], [10]. In ascidians by contrast, FGF is essential for neural induction while BMP inhibition does not seem to be involved [11], [12].

Comparative embryology within each of these groups and with vertebrates provides an outstanding opportunity to assess the diversity of regulatory strategies leading to a common shared body plan and to test models of gene regulatory network evolution proposed in other bilaterian groups [13], [14]. In this context, ascidians can be regarded as interesting chordate evolutionary outliers with unique developmental and genomic features. Their mode of development, based on small cell numbers and invariant cell lineages, diverges markedly from that found in vertebrates and amphioxus [15]. In addition, ascidians also display a fast rate of evolution with extensive genome rearrangements and compaction as well as gene losses [16], [17]. Ascidian genomes are thus very different from other chordate genomes (for example, synteny and ultra conserved elements conserved between vertebrates and amphioxus are not found in ascidians) [18], [19]. Finally, the high conservation of ascidian cell lineages throughout ascidian groups allows the comparison of genomically divergent ascidian embryos with a cellular level of resolution [20]–[22].

**Figure 1:**
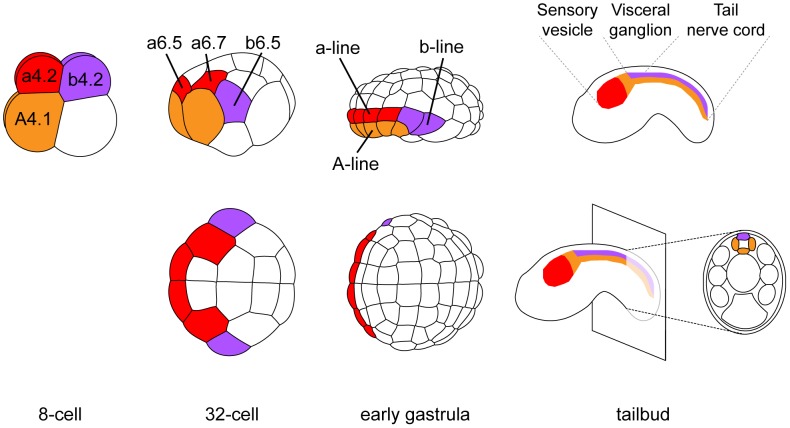
Cell lineages of the ascidian central nervous system. At each developmental stage, cells contributing to the central nervous system are colored according to their origin in the 8-cell stage embryo. a-line CNS (red) originates from anterior animal blastomeres (a4.2 pair) and forms the anterior sensory vesicle. A-line CNS (orange) originates from anterior vegetal blastomeres (A4.1 pair) and forms the posterior sensory vesicle, the visceral ganglion and the tail nerve cord (only the ventral and lateral parts for the latter two regions). b-line CNS (purple) originates from posterior animal blastomeres (b4.2 pair) and forms the dorsal part of visceral ganglion and tail nerve cord. Drawings for 8-cell to early gastrula stages: lateral view with animal to the top and anterior to the left (top row) and animal view with anterior to the left (bottom row). Drawings for tailbuds are lateral views with dorsal to the top and anterior to the left and a cross-section through the tail showing the four cells originating from two distinct lineages (A- and b-line).

**Figure 2:**
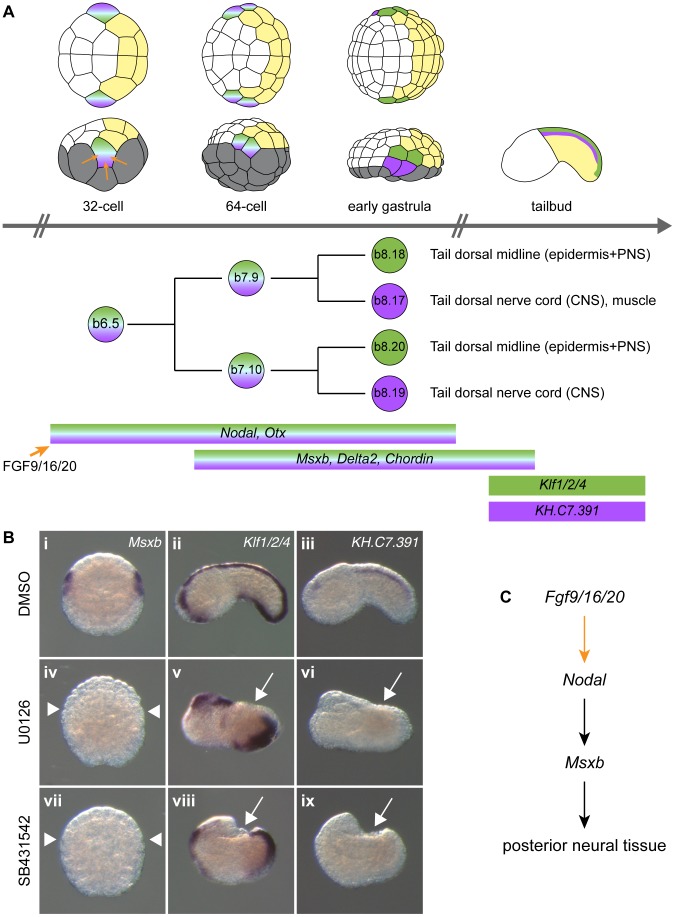
FGF and Nodal signaling are required for posterior neural tissue formation. A) Schematic representation of b6.5 lineage history with representation of embryos, cell lineage and gene expression at different stages. The different tissues and precursors are color coded: vegetal cells in grey, anterior (a-line) ectoderm in white, posterior (b-line) ectoderm in yellow, dorsal tail epidermis in green and dorsal tail nerve cord in purple. Embryos are in animal view (top row) or lateral view (bottom row) with anterior to the left. B) Expression of early and late b6.5 lineage markers when FGF-Erk and Nodal signaling pathways are disrupted. *Msxb* which is normally expressed in the four daughter cells of the b6.5 blastomere (Bi) is not expressed in U0126-treated (Biv) and SB431542-treated (Bvii) embryos. Animal views of *Msxb* at early gastrula stages (stages 10/11) (Bi, iv, vii). Schematic animal views of stage 10 embryos are depicted as insets in *Msxb* panels: anterior ectoderm in white, posterior ectoderm in yellow and gene expression in blue. Expression of *Klf1/2/4* is lost in tail dorsal midline for both treatments (Bv and Bviii). The dorsal tail nerve cord marker *KH.C7.391* is suppressed (Bvi and Bix). Lateral view with dorsal to the top and anterior to the left (Bii, iii, v, vi, viii and ix) at stage 19. Control DMSO-treated embryos (Bi-iii), U0126-treated embryos (Biv-vi) and SB431542-treated embryos (Bvii-ix). White arrows and arrowheads indicate sites with a loss of expression. C) Gene interactions revealed by loss-of-function data.

The dorsal hollow neural tube of the ascidian larva is composed of three morphologically distinct regions: the sensory vesicle anteriorly, the visceral ganglion and the tail nerve cord posteriorly (Figure 1). While there are still debates on their precise homology to vertebrate CNS domains, they are thought to be equivalent to fore/midbrain, hindbrain and spinal cord respectively [23], [24]. The ascidian CNS has a dual origin and specification logic (reviewed in [25]). Three separate lineages, named according to the founding blastomeres of the 8-cell stage embryo, form the ascidian CNS (Figure 1). The A-line neural lineage originates from vegetal blastomeres and gives rise to the posterior part of the sensory vesicle and to the ventral and lateral parts of both visceral ganglion and tail nerve cord. Ectodermal blastomeres give rise to the anterior part of the sensory vesicle (a-line) and to the dorsal part of the visceral ganglion and tail nerve cord (b-line). While A-line CNS is specified autonomously [26], a- and b-line are specified through neural induction by FGF9/16/20 secreted from the vegetal hemisphere at the 16- to 32-cell stage transition [11], [12], [27], [28]. Early target genes including *Otx*, *Nodal*, *Elk* and *Erf* are expressed at the 32-cell stage in all or part of the neural precursors (a6.5 and b6.5 blastomeres; Figures 1 and S2) where ERK signaling is active [11], [29], [30]. Interestingly, each of these precursors also contributes to the peripheral nervous system (PNS) following FGF9/16/20 induction [31], [32]. For example, the b6.5 blastomere gives rise to the dorsal midline of the tail epidermis, a neurogenic territory from which the epidermal sensory neurons of the PNS form (Figure 2A). Beside the requirement of *Otx* for anterior neural tissue formation [33] and the key role of *Nodal* in A-line CNS patterning and formation of the b6.5 derivatives [23], [29], [32], [34], [35], little is known for the function of these immediate target genes in neural fate acquisition or stabilization.

In order to gain insights into post-neural induction events, we focused our attention on the regulation of *Msxb* and *Delta2*, markers of the progeny of the b6.5 blastomeres. Both genes are expressed from the 64-cell stage (after neural induction) in the b6.5 progeny (b7.9 and b7.10 blastomere pairs; Figure 2A and [36], [37]) and are required for further specification and differentiation of these progenitors. *Msxb* is a marker of the entire b6.5 lineage until neurula stages, and is required for tail dorsal epidermal midline and dorsal nerve cord formation [23], [35]. *Delta2* is involved in the specification of epidermal sensory neurons within the epidermal midline [32], [38].

In this study, we show that FGF signaling is necessary and sufficient for b6.5 fate acquisition in posterior ectoderm. Downstream of FGF, *Nodal* is necessary for b6.5 fate. Although it cannot induce neural tissue on its own, it is sufficient to posteriorize FGF-induced neural tissue. This led us to search for other factors acting with *Nodal* downstream of FGF. We uncovered a critical function for the transient expression of *Otx* in posterior neural fate acquisition. Using this simple model of regulation, we were able to isolate b6.5 lineage specific enhancers for both *Msxb* and *Delta2*. We further show that this mode of regulation is shared with the distantly related ascidian *Phallusia mammillata*, strengthening our proposal that *Otx*, a well known regulator of anterior neural tissues in many metazoans, has been co-opted in ascidians for posterior nervous system formation.

## Results

### FGF signaling is necessary and sufficient for posterior ectodermal cells to adopt a b6.5 fate

Previous reports indicated that induced b6.5 fates are lost after abolition of FGF signaling [11], [28], [35]. We extended these results using a pharmacological inhibitor of FGF/MEK signaling (U0126), three early markers of b6.5 progeny (*Msxb*, *Delta2* and *Chordin*) and two tailbud markers of dorsal tail epidermis midline and dorsal nerve cord, *Klf1/2/4* and *KH.C7.391* respectively (Figures 2 and S1). MEK inhibition led to a conversion of neural b6.5 progenitors into epidermis as demonstrated by the loss of expression of all neural markers, coupled to the ectopic expression of the epidermal marker *Ap2-like2* at gastrula stages (Figure S1).

**Figure 3:**
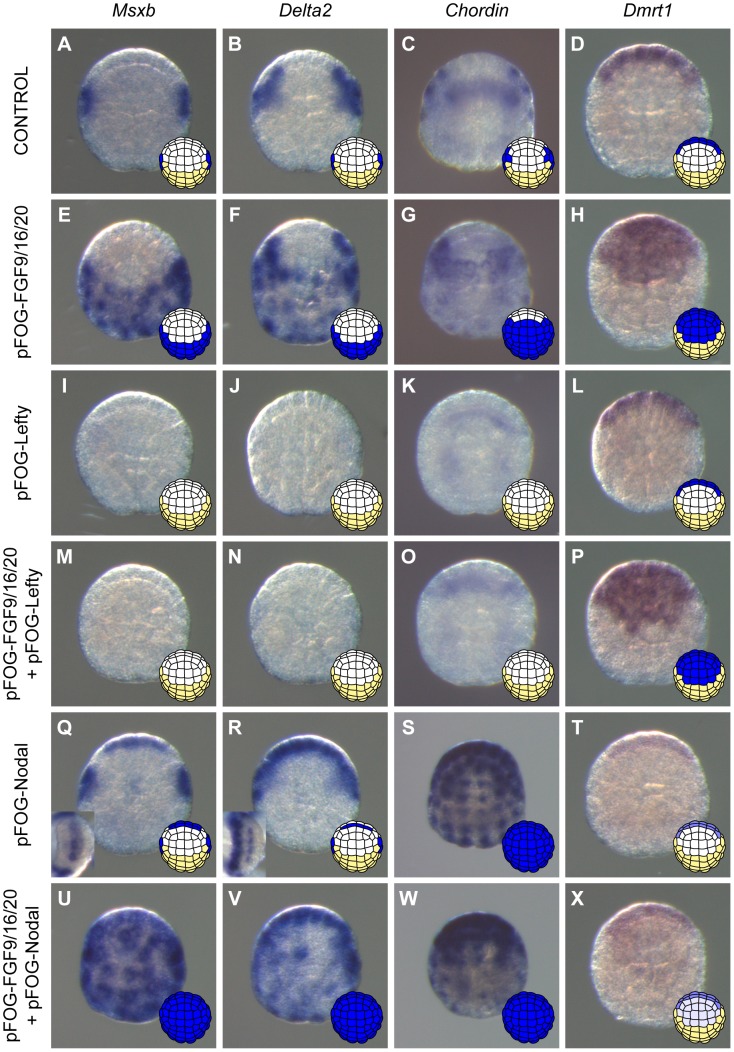
Nodal acts downstream of FGF to posteriorize induced neural tissue. Expression of posterior neural markers (*Msxb* (A), *Delta2* (B), and *Chordin* (C)) and of the anterior neural marker *Dmrt1* (D) in control embryos. FGF9/16/20 overexpression using the pFOG promoter *via* electroporation led to ectopic expression of *Msxb* (E) and *Delta2* (F) throughout posterior ectoderm, of *Chordin* (G) through most of the ectoderm except the anterior-most part and of *Dmrt1* (H) throughout anterior ectoderm at early gastrula stages (st. 10/11). These effects were suppressed by inhibition of Nodal signaling through Lefty overexpression (M-O) except for *Dmrt1* (P). Overexpression of Lefty alone inhibited posterior marker expression (I-K) but did not affect expression of the anterior marker *Dmrt1* (L). Overexpression of Nodal using the pFOG driver was sufficient to activate *Msxb* (Q) and *Delta2* (R) in the neural plate, and *Chordin* (S) throughout the ectoderm. Ectopic *Chordin* expression was stronger in anterior ectoderm than in posterior ectoderm possibly reflecting the difference in expression levels between anterior and posterior expressing cells in control embryos. *Dmrt1*expression was downregulated (T). Combined overexpression of FGF9/16/20 and Nodal led to ectopic activation of *Msxb* (U) and *Delta2* (V) in both anterior and posterior ectoderm. Under these conditions, *Chordin* was still expressed throughout the ectoderm but at weaker levels (W). Overexpression of Nodal downregulated ectopic activation in anterior ectoderm of *Dmrt1* induced by FGF9/16/20 (X). Animal view with anterior to the top for all except insets in Q and R that show neural plate view with vegetal side to the left. For each panel a schematic animal view of stage 10 embryos depicts anterior ectoderm in white, posterior ectoderm in yellow and gene expression in blue.

Previous reports indicated that activation of the FGF pathway in explanted ectodermal precursors leads to the induction of neural fate in cells normally fated to form epidermis, with different neural fates achieved in a-line and b-line blastomeres [11], [12], [27], [32]. We confirmed that this was also the case in whole embryos. We treated whole embryos either with recombinant FGF protein from the 16-cell stage or overexpressed FGF9/16/20 by electroporation using the pFOG driver (expressed from the 16-cell stage throughout the entire ectoderm [39]). As expected, the epidermis marker *Ap2-like2* was strongly down-regulated throughout the ectoderm (data not shown). The posterior neural markers *Nodal*, *Msxb* and *Delta2* were ectopically expressed throughout the posterior ectoderm (b4.2 lineage or b-line ectoderm), and the anterior neural marker *Dmrt1* was activated throughout the anterior ectoderm (a4.2 lineage or a-line ectoderm) (Figures 3A-H and S2). *Chordin*, which is normally expressed in the progeny of b6.5 as well as in a8.26 and a8.28 blastomere pairs (Figure 3C), was expressed throughout the posterior ectoderm and in part of the anterior ectoderm in response to ectopic FGF treatment (Figures 3C and 3G).

*Nodal* activation at the 32-cell stage was a likely direct consequence of FGF signaling. FGF treatment activated *Nodal* ectopic expression in the presence of protein synthesis inhibitor (Figures S2), suggesting the absence of a transcriptional relay. In addition, a previously identified b6.5-specific *Nodal* enhancer has the same regulatory logic as the FGF-responsive enhancer of the direct FGF target gene *Otx* [30]. *Msxb*, *Delta2* and *Chordin* are more likely to be indirect targets of FGF as they are activated later at the 64-cell stage.

In the following sections, we will precisely define the regulatory interactions between FGF, *Nodal*, *Otx*, *Msxb*, *Delta2* and *Chordin* in the b6.5 lineage.

### Nodal signaling posteriorizes FGF-induced neural tissue

To determine the function of Nodal during b6.5 fate acquisition, we blocked the function of its receptor with the pharmacological inhibitor SB431542 or overexpressed the Nodal antagonist Lefty in the ectoderm using electroporation. Both perturbations led to a loss of expression of *Msxb*, *Delta2* and *Chordin* in b-line neural lineage at gastrula stages (Figures 2B, 3I-K and S1). At later stages, expression of the dorsal tail nerve cord marker *KH.C7.391* was lost, as was the dorsal expression of the tail midline marker *Klf1/2/4* (Figure 2B). This altered genetic program was similar to that obtained in response to FGF inhibition, suggesting that Nodal acts downstream of *Fgf9/16/20* in b-line neural specification (Figure 2C). Consistent with this, FGF-induced ectopic activation of *Msxb*, *Delta2* and *Chordin* was suppressed by Lefty overexpression (Figure 3M-O). Nodal was however not the sole mediator of FGF action, as its inhibition was not sufficient to convert the b6.5 progeny into epidermis, marked by *Ap2-like2* expression (Figure S1).

We next overexpressed *Nodal* throughout the ectoderm using the pFOG driver and analyzed marker expression in the a- and b-line ectoderm. Ectopic expression of *Chordin* was observed throughout the ectoderm (Figure 3S), independently of the FGF induction status of the cells. Ectopic *Chordin* expression was stronger in a-line ectoderm, possibly reflecting the stronger levels detected in a8.26 and a8.28 blastomeres compared to b6.5 progeny in control embryos (Figure 3C). By contrast, we did not detect ectopic activation of *Msxb* and *Delta2* in posterior (b-line) ectoderm (Figure 3Q,R). However, anterior neural tissue precursors (a6.5 lineage) ectopically expressed these two genes (Figure 3Q,R) and had reduced *Dmrt1* expression (Figure 3T). These data indicate that anterior neural precursors adopted a posterior identity in response to Nodal expression. Consistent with these observations, co-electroporation of pFOG-FGF9/16/20 and pFOG-Nodal, led to the induction of posterior neural tissue in anterior ectoderm, demarcated by the ectopic activation of both *Msxb* and *Delta2* and by the repression of *Dmrt1* (Figure 3U,V and X).

The results of this section indicate that *Nodal* alone is required, though not sufficient, to induce neural tissue and that it can posteriorize FGF-induced neural tissue. Interestingly, expansion of the anterior neural marker *Dmrt1* to posterior b-line territories was not observed following Nodal signaling inhibition in either wild type or FGF-induced contexts (Figure 3L, P). These results are consistent with the presence of a *Nodal*-independent factor necessary for *Dmrt1* expression and anterior neural fate acquisition in a-line ectoderm [40], [41] (see discussion).

In summary, three genes expressed downstream of FGF in the b6.5 progeny show different requirements regarding Nodal signaling: *Chordin* can be activated in the entire ectoderm while *Msxb* and *Delta2* are positive targets of Nodal solely in FGF-induced neural cells.

### *Otx* is required for posterior neural tissue formation

The conversion of a6.5 anterior neural precursors into posterior neural fate upon ectopic activation of Nodal signaling (Figure 3 Q, R) suggests that posterior neural fates may result from the cooperation of Nodal with another FGF-target. *Otx* is a conspicuous candidate since it is expressed in all neural precursors downstream of FGF signaling (Figure S2) and is coexpressed with Nodal in posterior neural precursors marked by *Msxb* and *Delta2* expression [11], [27] (Figures 2, 3 and S2).

**Figure 4:**
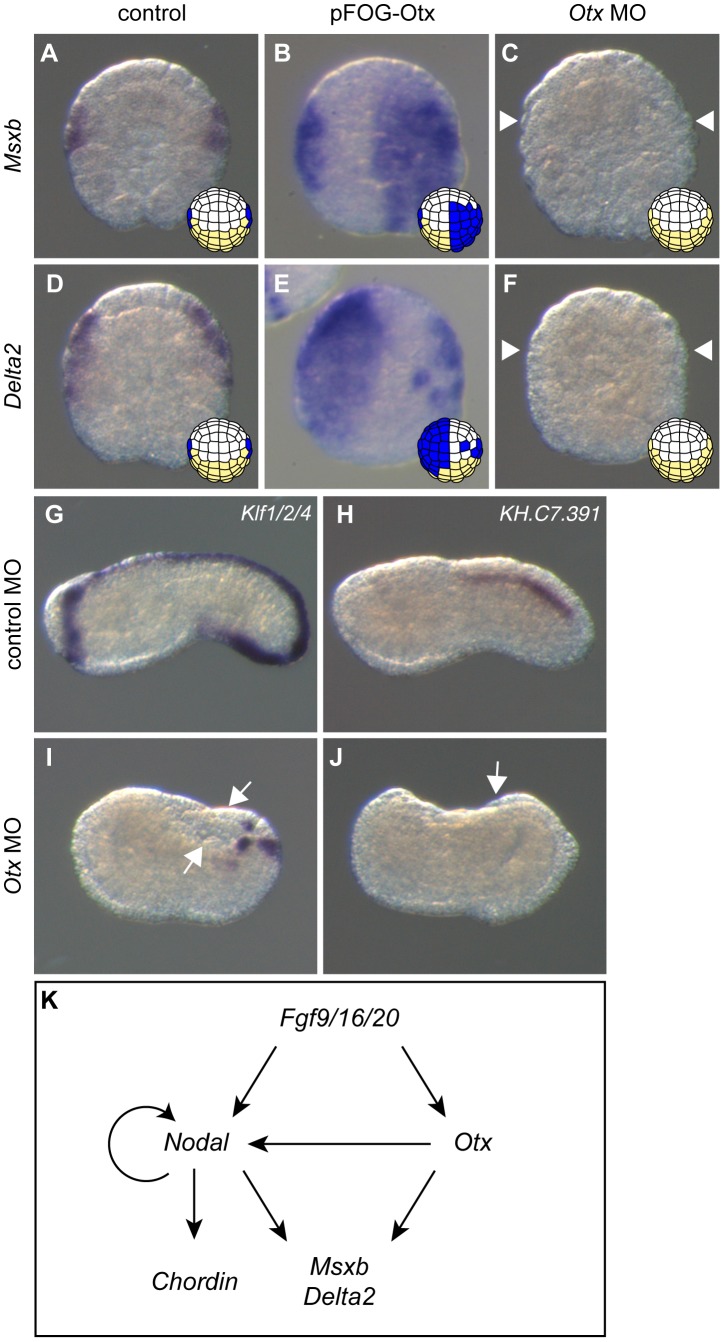
*Otx* is required for posterior neural tissue formation. Overexpressing Otx in the ectoderm using the pFOG driver is sufficient to activate *Msxb* (B) and *Delta2* (E) compared to control embryos (A, D) at stage 10. Upon injection of the *Otx* MO, *Msxb* (C) and *Delta2* (F) expression is abolished at stage 10. The dorsal expression of the tail midline marker *Klf1/2/4* is lost except in the posterior-most and ventral regions (I). The dorsal nerve cord marker *KH.C7.391* expression is also suppressed (J). Control MO-injected embryos at stage 10 (A, D) and stage 19 (G, H). Animal view with anterior to the top (B, D-F). Vegetal view with anterior to the top (A and C). Lateral view with dorsal to the top, anterior to the left (G-J). White arrows and arrowheads indicate sites with a loss of expression. (K) Summary of gene interactions reported in this study and from previous studies [11], [29], [30], [35].

We first tested the requirement of *Otx* in b6.5 fate acquisition by injecting a specific translation-blocking morpholino antisense oligonucleotide (MO). *Otx* morpholino injection led a full loss of *Msxb* and *Delta2* expression at stage 10 (Figure 4C, F). The resulting embryos displayed gastrulation and neurulation defects reminiscent of FGF or Nodal signaling inhibition. The tail midline marker *Klf1/2/4* was strongly affected (Figure 4I). Dorsal tail epidermis midline staining originating from b6.5 was abolished while posterior-most staining (originating from b6.6 lineage) was maintained. Ventral midline expression was also kept but the domain of expression appeared reduced in size. Dorsal tail nerve cord did not form either as revealed by the loss of the marker *KH.C7.391* (Figure 4J). We obtained similar results by overexpressing a dominant negative form of Otx, OtxHDenR (a fusion protein between the Otx homeodomain and the repressor domain of Engrailed) [42] in the ectoderm (Figure S4). The phenotypes appeared milder probably because OtxHDenR was only targeted to the ectoderm and because of the mosaic inheritance of the transgene introduced by electroporation. In addition, we observed that expression of the epidermal marker *Ap2-like2* was unchanged following overexpression of OtxHDenR (Figure S4). Similarly to what has been observed for Nodal inhibition, b-line neural lineage did not form neural tissue upon Otx loss-of-function but did not form epidermis either.

We next tested the effect of Otx overexpression using the pFOG driver. Although we expected that *Otx* would need to cooperate with *Nodal* to activate *Msxb* and *Delta2*, *Otx* overexpression was sufficient to activate both of these latter genes throughout the ectoderm (Figure 4B, E). When we overexpressed simultaneously *Otx* and *Nodal* throughout the ectoderm, we simply observed an addition of each molecule effect with no increase in the number of embryos ectopically expressing *Msxb* and *Delta2* in the ectoderm (data not shown). To better understand these results, we further explored possible transcriptional interactions between *Nodal* and *Otx* that may control maintenance of their expression following the initial induction by FGF (Figure S2). We detected robust activation of *Nodal* expression at the 64-cell stage when *Otx* was ectopically expressed (Figure S3Aii). Accordingly, *Nodal* expression was repressed by the overexpression of OtxHDenR (Figure S3Aiii). This interaction between *Otx* and *Nodal* was not reciprocal, since *Otx* expression was not changed upon modulation of Nodal signaling (Figure S3Avi, vii). Nodal signaling inhibition also prevented *Nodal* expression (Figure S3Aiv), suggesting the existence of an autoregulatory loop on *Nodal* similarly to what has been described in vertebrates [43]. The ectopic activation of *Msxb* and *Delta2* in the ectoderm by Otx overexpression did not require the activation of Nodal, as overexpression of Lefty did not significantly block Otx effect (Figure S3B). By contrast, Nodal-mediated ectopic expression of *Msxb* and *Delta2* in anterior neural precursors was inhibited by OtxHDenR overexpression (Figure S3C).

These data demonstrate that *Otx* is an essential regulator of b6.5 lineage derived posterior neural tissue formation. Figure 4K provides a schematic representation of the gene regulatory network acting downstream of FGF in b-line ectoderm.

### The genomic hardwiring of *Msxb* and *Delta2* regulation

**Figure 5:**
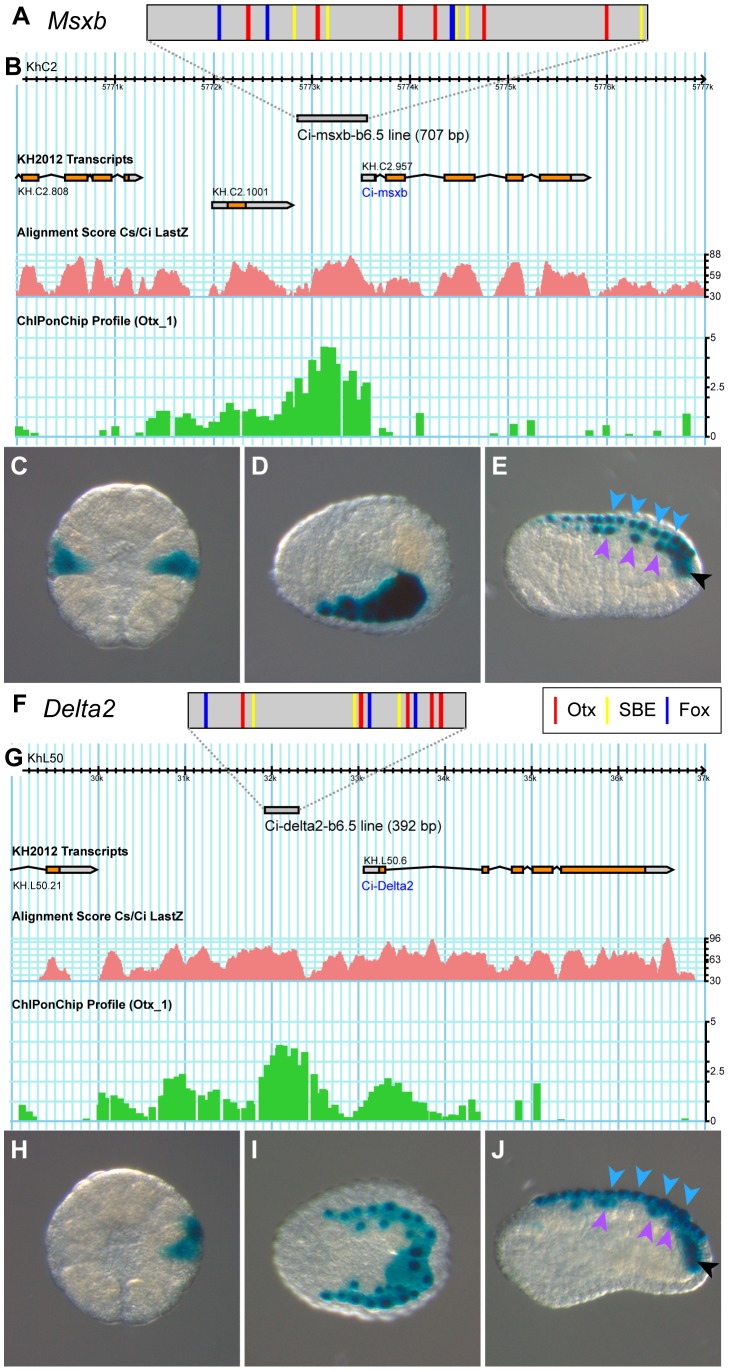
The b6.5 line enhancers of *Msxb* and *Delta2*. Schematic organization of tested enhancers depicting putative Otx (GATTA) (red bars), Fox (AAACA) (blue bars) binding sites and SBEs (AGAC) for *Msxb* (A) and *Delta2* (F). Genomic browser view of gene loci with gene models (*Msxb*: KH.C2.957, *Delta2*: KH.L50.6), tested enhancers (grey bar), alignment profile of *C. intestinalis* and *C. savignyi* genomic sequences (pink) and ChiP-on-Chip data (green) [50] for *Msxb* (B) and *Delta2* (G) (extracted from the Aniseed genome browser: http://www.aniseed.cnrs.fr/fgb2/gbrowse/ciona_intestinalis/ [70], and from the Ghost genome browser http://ghost.zool.kyoto-u.ac.jp/cgi-bin/gb2/gbrowse/kh/ [71]). Representative pictures for X-gal staining of electroporated embryos with the “msxb-b6.5 line” enhancer at stage 10 (C), stage 14 (D) and stage 16 (E), and with the “delta2-b6.5 line” enhancer at stage 10 (H), stage 14 (I) and stage 18 (J). Arrowheads indicate dorsal midline epidermis (blue), dorsal nerve cord (purple) and secondary muscle (black). Vegetal view, anterior to the top (C, H). Dorsal view, anterior to the left (D, I). Lateral view, dorsal to the top and anterior to the left (E, J). Additional staining was also observed in mesenchymal cells, a tissue highly permissive to transcriptional assays in *Ciona* [42].

We next used the above functional evidence to isolate *cis*-regulatory DNA regions responsible for neural marker expression in the b6.5 lineage. We reasoned that the enhancer responsible for b6.5 lineage expression should integrate both Otx and Nodal inputs. Nodal is a ligand which controls gene expression through the activation of the Smad2/3 nuclear effector. A Smad2/3/Smad4 complex can directly bind DNA with low affinity through poorly defined GC rich regions or through (C)AGAC Smad Binding Element (SBE) consensus sequences [44]. However, high affinity binding is usually achieved through association with a DNA binding cofactor. In several instances, Fox transcription factors have been shown to fulfill this function [44]–[46]. We consequently searched the *Msxb* locus for the co-occurrence of Otx and Fox/Smad binding sites. We selected the core consensus sequences GGATTA for Otx, TGTTT for Fox from the Jaspar database [47], and AGAC for Smad [44]. We searched for regions enriched in Otx-, Fox- and Smad- core binding site motifs by first scanning, in *Ciona intestinalis* type A [48], the 50 kb genomic region that includes *Msxb* up to its two flanking genes. We arbitrarily chose a 300 bp window and found 15 regions that contained at least one of each motif. To reduce the number of candidates we increased the stringency by increasing the number of the least frequent site, which is Otx. We chose a more degenerate site for this additional motif, GATTA, as in [42]. Adding one or two GATTA motifs yielded 7 and 4 candidate regions, respectively. We focused on the latter 4 regions and searched whether the *Ciona savignyi* orthologous regions harbored a similar combination of binding sites using Vista suite [49]. A single region matched this criterion and was named “msxb-b6.5 line” according to its enhancer activity (see below) (Figure 5). This region is located just upstream of *Msxb* on a peak of conservation and contains 6 putative Otx, 5 putative Fox binding sites and 6 putative SBEs (Figures 5A, B and S5). This region falls within a region bound *in vivo* by Otx at early gastrula stages as revealed by ChIP-on-Chip experiment (Figure 5B) [50].

We amplified this 707 bp fragment from *C. intestinalis* type B genomic DNA. The sequence obtained is very similar to the reference type A sequence but contains only 4 Fox binding sites and 5 SBEs (Figure S5). Placed upstream of the minimal promoter of *Fog* and the reporter gene *LacZ* [39], [51], this fragment drove transcription throughout b6.5 derivatives from the early gastrula stage (Figure 5C-E and Table S1). Thus, searching for enrichment in Otx, Fox and Smad putative binding sites in conserved non-coding genomic DNA was sufficient to isolate a region, which binds Otx *in vivo* at the early gastrula stage and is transcriptionally active in posterior neural precursors.

The same logic led to the identification of a *Delta2* enhancer active in the b6.5 lineage. A single genomic region at the *Delta2* locus harbored a combination of Otx, Fox and SBE sites within 300 bp in both *C. intestinalis* and *C. savignyi* and was named “delta2-b6.5 line” (Figure 5). This 392 bp long region is located within 2 kb upstream of *Delta2*, harbors a strong level of conservation, contains 5 Otx sites, 3 Fox sites and 3 SBEs; and is bound *in vivo* by Otx (Figures 5F-G and S6). When electroporated in *C. intestinalis* embryos it drove expression in b6.5 derivatives from early gastrula stages (Figure 5H-J and Table S1).

Overall, these results indicate that *Msxb* and *Delta2* share similar regulatory motifs in their enhancers.

### *Msxb* enhancer activity relies on Otx, Fox and Smad binding sites

**Figure 6:**
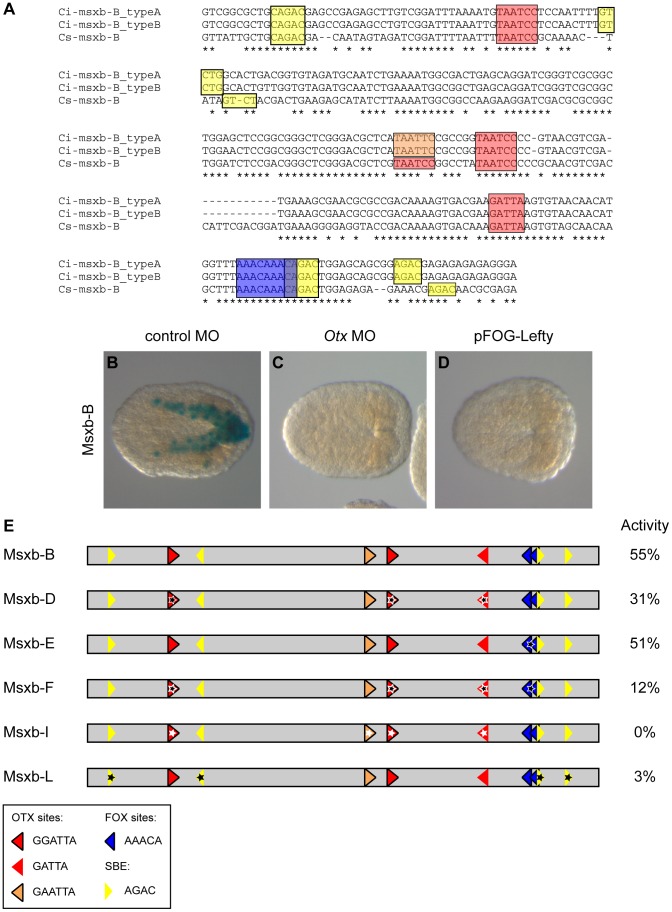
Otx, Fox and Smad putative binding sites control msxb-B enhancer activity. A) Alignment of msxb-B sequences from *C. intestinalis* type A, *C. intestinalis* type B and *C. savignyi*. Putative transcription factor binding sites are in colored boxes as follows: canonical Fox (AAACA) in dark blue, canonical Otx (GATTA) in red, non-canonical Otx (GAATTA) in orange and SBE (AGAC) in yellow. B) The msxb-B enhancer is active in b6.5 derivatives as revealed by X-gal staining on late gastrula. Its activity is abolished upon injection of the *Otx* MO (C) or overexpression of Lefty (D). E) Schematic view of msxb-B enhancer and its mutated versions. Putative transcription factor binding sites position and orientation are represented by colored arrows with the same color code as in (A). Mutations are depicted by stars. The precise mutations are described in the main text and in figure S8. Transcriptional activity of the different enhancers was measured as the percentage of embryos with staining in the b6.5 derivatives at late gastrula stages (stage 14) (Table S1).

We next assayed the relative contribution of Otx, Fox and Smad binding motifs to enhancer activity in the b6.5 lineage, focusing on the “msxb-b6.5 line” enhancer. Progressive shortening of this region on both sides (Figure S7 and Table S1) identified an active 273 bp long fragment (msxb-B) containing 3 Otx binding motifs, 2 overlapping Fox binding motifs and 4 Smad motifs (Figure 6A-B). This fragment was still active in inverted orientation (Msxb-B-inv), as expected from an enhancer (Figure S8B). Msxb-B enhancer activity was abolished when the *Otx* morpholino was injected and when Lefty was overexpressed (Figure 6B-D).

Simultaneous mutation of the 3 Otx sites through a single nucleotide modification in the core (GATTA = >GcTTA) (construct Msxb-D) led to a partial loss of activity (Figure 6E). Since activity was not completely suppressed, we looked for potential Otx binding motifs with altered core sequence. Interestingly, we found a GAATTA motif that corresponds to a canonical GGATTA sequence in *Ciona savignyi* (Figure 6A). Simultaneous mutation of this and the 3 canonical Otx sites (GNATTA = >GNcgTA) (construct Msxb-I) led to a complete loss of activity.

We next mutated the 4 conserved Smad Binding Elements (AGAC = >ctAC) and found these sites to be essential for Msxb-B activity (Msxb-L construct; Figure 6A, E).

We finally mutated the Fox sites. Two AAACA sites overlap in the AAACAAACA sequence (Figure 6A). We generated either a single nucleotide change that matches in the core of each Fox site (AAACgAACA, Msxb-E) or a single nucleotide change in each core (AAgCAAgCA, Msxb-H) (Figures 6E and S8). These mutations did not affect enhancer activity. Additional mutation (AACA = >AgCA, Msxb-G) of the three more degenerate AACA consensus found in the sequence, but not conserved in *C. savignyi*, also had no effect (Figure S8). We then tested the effect of mutating Fox sites in the sensitized context of the Msxb-D element where 3 Otx sites are mutated and where activity is decreased. The Msxb-F fragment (3 Otx sites mutated, 2 canonical Fox sites mutated) displayed a further reduction in activity (Figure 6E), suggesting that Otx and Fox sites may work together to control Msxb-B activity.

Mutational analysis indicates that *Msxb* regulation through the Msxb-B enhancer may involve putative Fox binding sites and requires the presence of putative Otx and Smad binding sites to be transcriptionally active in b6.5 derived cells.

### A conserved regulatory logic across distantly related ascidian genera

**Figure 7:**
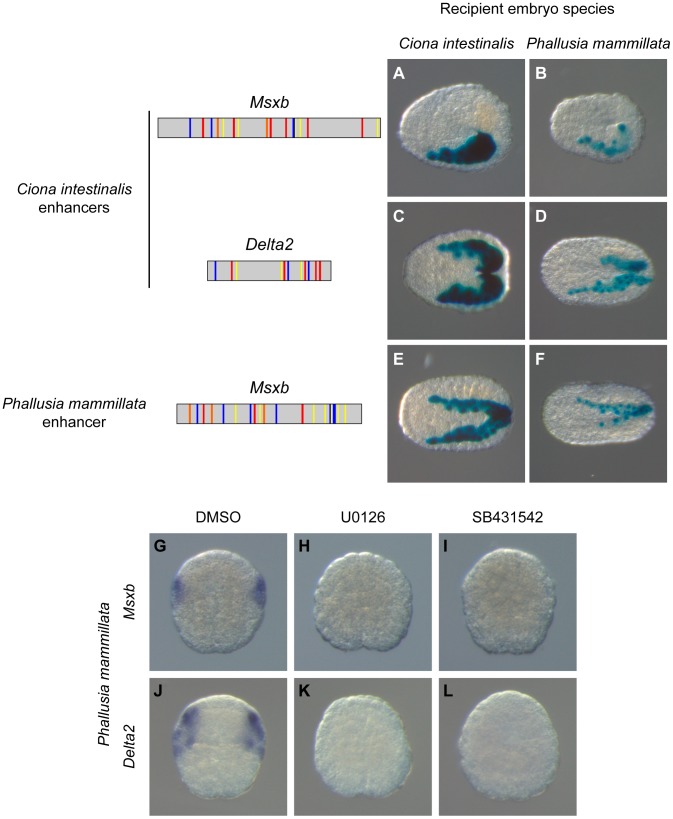
A shared regulatory logic in *Ciona intestinalis* and *Phallusia mammillata*. Schematic organization of tested enhancers with the same color code used in figures 4 and 5. Reporter gene activity is detected by X-gal staining after electroporation of “Ci-msxb-b6.5 line” enhancer (A, B), “Ci-delta2-b6.5 line” enhancer (C, D) and “Pm-msxb-b6.5 line” enhancer (E, F) into *C. intestinalis* (A, C, E) or *P. mammillata* (B, D, F) embryos. Transcriptional activity of the different enhancers, measured as the percentage of embryos with staining in the b6.5 derivatives, is detailed in Tables S1 and S2. In *Phallusia mammillata* embryos, *Msxb* (G) and *Delta2* (J) are expressed in b6.5 derivatives. This expression is abolished upon inactivation of the FGF/MEK (H, K) or Nodal (I, L) signaling pathway. Dorsal view with anterior to the left (A-F). Animal view, anterior to the top (G-L).

We tested the transcriptional activity of the *Ciona Msxb* and *Delta2* enhancers that we identified in a distantly related and genomically divergent ascidian, *Phallusia mammillata*. When each construct was electroporated in *P. mammillata* embryos, we detected LacZ activity in dorsal tail epidermis midline, dorsal nerve cord and secondary muscle, the same territories that are stained in *C. intestinalis* (Figure 7B, D and Table S2). These results suggest that the regulatory logic of these enhancers is interpreted in the same way in *C. intestinalis* and *P. mammillata* embryos. The similar enhancer activity between these two species possibly reflects conservation of the combination of transcription factors, the *trans*-regulatory logic, acting upstream of *Msxb* and *Delta2*. We further tested this possibility by determining the expression patterns of *Msxb* and *Delta2* in *P. mammillata* by *in situ* hybridization (Figure S9). We observed that both genes are activated in the b6.5 lineage at the 64-cell stage (b7.9 and b7.19 blastomeres) like the *C. intestinalis* orthologous genes. This expression was abolished when inhibitors of the FGF/MEK (U0126) and Nodal (SB431542) signaling pathways were applied to the embryos (Figure 7G-L).

These results led us to search for enhancers regulating *Msxb* expression in *P. mammillata*. Employing the same strategy we used for *C. intestinalis* genes, we searched the *Pm-Msxb* locus for regions enriched in Otx, Fox and Smad binding motifs and conserved in the sister species *Phallusia fumigata*. We isolated a 587 bp fragment containing 6 Otx, 7 Fox binding motifs and 7 SBEs and located just upstream of *Pm-Msxb* (Figure S10). This fragment, “Pm-msxb-b6.5 line”, whose sequence could not be aligned with that of “Ci-msxb-b6.5 line”, was active in b6.5 derivatives when electroporated in *P. mammillata* (Figure 7F) or *C. intestinalis* (Figure 7E) embryos (Tables S1 and S2). Therefore, the functional knowledge acquired in *C. intestinalis* was sufficient to isolate an active enhancer with expected activity in another species, *P. mammillata*.

## Discussion

We have shown that *Nodal* and *Otx*, directly activated by the neural inducer FGF9/16/20 at the 32-cell stage, are required for posterior neural fate acquisition. We propose that these two genes act in concert to promote the activation of *Msxb* and *Delta2* at the 64-cell stage. This simple model allowed us to isolate an enhancer for each gene containing Otx, Fox and Smad binding sites and active in the posterior neural lineage. We also showed that this regulatory logic is conserved, in a distantly related ascidian species *Phallusia mammillata*, in spite of extensive sequence disparity.

### Molecular mechanisms downstream of neural induction for neural fate acquisition

FGF-triggered neural induction in *Ciona* appears, at first glance, to be a simple inductive process whereby two blastomeres (a6.5 and b6.5) receive a signal from the vegetal hemisphere and adopt a neural fate instead of an epidermal fate (Figures 1 and 2A). However, this event is tightly controlled: ectodermal cell competence is regulated [39], [40], embryo geometry [52] and various signaling pathways [41] also control the response of the ectoderm to the inducer.

We have shown that three FGF-dependent genes expressed in the b6.5 progeny from the 64-cell stage show differential regulation by Nodal signaling. *Chordin* is probably directly regulated by Nodal while *Msxb* and *Delta2* need additional inputs from Otx. Our data provide additional connections and genomic hardwiring to a previously described network [35]. The network of genes regulating posterior neural fate is not linear and includes several regulatory loops (Figure 4K). FGF activates at least two direct target genes, *Otx* and *Nodal*, at the 32-cell stage, which collectively regulate secondary targets (*i.e. Msxb* and *Delta2* at the 64-cell stage). Moreover, the regulation that we have uncovered involves a transcription factor and a signaling molecule that are expressed in the same cells. It is possible that this configuration allows very tight transcriptional control in a lineage-restricted manner using autocrine signaling. Finally, we have uncovered additional interactions that most likely maintain gene expression in a lineage-restricted manner following initial activation. For example, maintaining *Nodal* expression in the b6.5 progeny following FGF induction is apparently controlled both by *Otx* and *Nodal* itself (Figures S3 and 4K).

The actual mode of concerted regulation of *Msxb* and *Delta2* by Otx and Nodal at the molecular level will need further investigation. We have proposed that the signaling molecule Nodal uses a Fox factor as a nuclear effector [44], [53]. This hypothesis led us to isolate three enhancers active in the b6.5 lineage. However, it is very likely that omitting Fox sites in our enhancer search would have led to the same outcome since Fox consensus sites (AAACA) are probably very abundant in the AT-rich ascidian genomes. Nevertheless, we observed that two overlapping Fox sites (AAACAAACA) are present in *Msxb* enhancers from both *C. intestinalis* and *P. mammillata* (Figures S5 and S10). However, mutation of these sites in “Ci-msxb b6.5 line” enhancer was silent unless some Otx sites were also mutated (Figure 6). The *C. intestinalis* genome encodes 29 predicted Fox factors whose expression pattern during early development has been determined [37], [54], but the number of candidate Fox factors (expressed in the b6.5 lineage or maternally provided) is beyond the scope of the current study. Although we cannot exclude the involvement of Fox factors in *Msxb* and *Delta2* regulation, we would favor an alternative scenario explaining the concerted action of Otx and Nodal. We have shown that Smad Binding Elements (SBEs) are essential for msxb-B enhancer activity, and the active enhancers that we have isolated contain at least three SBEs. We could thus conceive that Otx itself serves as a co-factor for Nodal signaling and that it would interact directly with activated Smad2/3 on the enhancer to promote transcriptional activation.

Besides activating secondary FGF targets, the function of direct FGF targets is an opened question. Epidermal *versus* neural fate decision is primarily controlled by FGF signaling. We have shown that inhibition of FGF, Nodal or Otx function abolishes b-line neural fate. However, contrary to the inhibition of FGF, blocking Nodal or Otx function does not lead neural precursors to adopt the alternative epidermal fate (Figures S1 and S4). These observations can be explained by two non-exclusive hypotheses: epidermis fate inhibition is achieved directly upon reception of FGF signaling or several direct FGF targets contribute to epidermis repression. In particular, in addition to *Otx* and *Nodal*, genes such as *Elk* and *Erf* are expressed in neural progenitors and are likely direct FGF targets [30], but their function has not been determined.

Following their activation at the 64-cell stage in the b7.9/10 blastomeres, *Msxb* and *Chordin* remain expressed in all daughter cells (until mid-gastrula stages) but *Delta2* expression becomes restricted in b8.18/20 blastomeres, precursors of the dorsal tail midline epidermis (Figure 2). This change in expression correlates with and may be involved in the fate restriction that occurs at early gastrula stages. This event is crucial since it separates central nervous system (dorsal nerve cord) and peripheral nervous system (dorsal tail midline epidermis) precursors. A similar CNS *versus* PNS segregation occurs at the same time in the anterior part of the embryo and involves FGF signaling [55]. While *Msxb* is essential for the formation of both dorsal tail epidermis midline and dorsal nerve cord [23], [35], the role of the two other genes remains to be investigated.

### Otx and Nodal in chordate posterior neural tissue formation

Otx is a transcription factor expressed in the anterior nervous system, and which participates to anterior neural patterning in many bilaterians [56], [57]. In ascidians, a similar role has previously been ascribed to this gene in two distantly related species *Ciona intestinalis* and *Halocynthia roretzi* [21], [27], [33], [35], [42], [58]. The additional involvement of *Otx* in posterior neural tissue formation that we describe in the present study is rather unexpected. However, the function of *Otx* that we have addressed corresponds to a very early phase of its dynamic expression. *Otx* has been shown to be a direct target of FGF signaling at the 32-cell stage [11]. The expression is transient (from the 32-cell stage to the 112-cell stage) in both anterior (a6.5 lineage) and posterior (b6.5 lineage) neural tissue precursors and precedes a new and massive expression only in the anterior neural plate (from early gastrula stages). This early phase marks neural induction in both ascidian species studied [11], [59], [60]. While the onset of expression of *Otx* homologs in vertebrates may be broader than the prospective anterior central nervous system [61], there is no report, to our knowledge, of participation of *Otx* genes in posterior nervous system formation. We consequently propose that Otx has been co-opted in ascidians for posterior neural tissue specification. Whether this co-option is unique to ascidians will await more functional data in invertebrate deuterostomes.

We have shown that Nodal is required for posterior neural tissue formation and that Nodal can posteriorize FGF-induced neural tissue. Interestingly, Nodal signaling is also involved in posterior neural tissue formation in vertebrates [62]–[64]. However, this is most likely indirect through the control of mesoderm specification and patterning. Nodal signaling is rather thought to be an anti-neural pathway whose activity needs to be shut down for neural fate acquisition [65], [66]. Our study shows that the function of Nodal signaling in ascidians is different from vertebrates: Nodal is not incompatible with neural fate and it can directly posteriorize neural tissue.

In *Ciona*, *Nodal* expression in posterior neural precursors is the result of differential competence of animal blastomeres to respond to FGF. This competence is controlled by *FoxA-a*, expressed in anterior blastomeres [35], [40], [41]. When FoxA-a function is abolished, anterior neural ectoderm adopts a posterior identity and ectopically expresses *Nodal* and *Delta2*. A phenotype similar to what we observed for Nodal ectopic misexpression. However, Nodal is not the only factor involved in posterior identity definition. When we blocked Nodal function, posterior neural precursors did not adopt an anterior identity. This result suggests that either expression of *FoxA-a* is necessary for anterior identity definition and/or that additional factor(s) control posterior identity redundantly with *Nodal*. It will be interesting to probe the involvement of other signaling pathways (Wnt, FGF and retinoic acid) that are also major regulators of posterior neurectoderm formation in vertebrates [4].

### A conserved regulatory signature in ascidians

Based on the combined regulation by Otx and Nodal, we were able to isolate enhancers containing putative Otx, Fox and Smad binding sites that control expression in the posterior neural lineage for two co-expressed genes. Interestingly, the “Ci-msxb-b6.5 line” enhancer is also active in anterior neurectoderm at tailbud stages (Figure S11) where several enhancers with an Otx signature have been described to be active [42]. This raises questions that will need further investigation. Are the same Otx-regulated enhancers re-used in different territories at different stages? Does the fragment we tested contain two distinct abutting or partially overlapping enhancers? These enhancers could consequently be the means for Otx co-option in posterior neural tissue. Finally, is Nodal signaling involved in later steps of anterior neurectoderm formation in *C. intestinalis*?

We have extended our study through cross-species transcriptional assay in two divergent ascidian species. Since *Otx* and *Nodal* display conserved expression in the b6.5 blastomeres in both *C. intestinalis* and *H. roretzi* [27], [29], [58], [67], it is very likely that they are also expressed in b6.5 in *Phallusia mammillata*, a species more closely related to *C. intestinalis*. This hypothesis can explain why we found conserved activity when *C. intestinalis* enhancers were tested in *P. mammillata* embryos. Importantly, we found that *Msxb* and *Delta2* from *P. mammillata* are expressed under the control of FGF and Nodal signaling pathways in b-line neural precursors. Together with the isolation of an active enhancer for *Pm-Msxb*, these results strongly support that gene regulation is also conserved. We have tried to extend our comparison to *Pm-Delta2* by testing several elements containing consensus Otx, Fox and Smad binding sites, but these elements were not active in posterior neural tissue precursors (data not shown). This can be explained by subtle changes in gene regulation or most likely by an incomplete understanding of the regulatory logic to be able to predict a functional enhancer (for example the tested elements had fewer Otx sites compared to the three active enhancers previously isolated). Interestingly, the *Msxb* enhancers that we isolated from each species do not show sequence conservation, they are not alignable. This is a general trend that has been observed by comparing ascidian genomes [21], [22]; mainly coding sequences retain sequence conservation and there is poor synteny conservation. This indicates that these genomes have largely diverged and underwent extensive reshuffling. This offers an excellent situation to probe enhancer evolution and transcription factor binding site turnover in genomes that control development of very similar embryos [20].

## Materials and Methods

### Embryo obtention and manipulation

*Ciona intestinalis* type B were provided by the Centre de Ressources Biologiques Marines in Roscoff. *Phallusia mammillata* were collected by diving in the Port-Vendres and Sète harbors, or collected from fishermen trawling in the Banyuls-sur-mer area. *C. intestinalis* embryology was performed as described in [32]. Staging was described according to [68]. *P. mammillata* embryos were handled the same way as *Ciona* except dechorionation was performed on unfertilized eggs for around 40 min with 0.1% trypsin and 0.5% sodium thioglycolate acid raised to basic pH by NaOH addition. Electroporation was performed as described [32] with the following modification: a single pulse of 25V for 32 ms (*C. intestinalis*) or 25 to 37V for 32 ms (*P. mammillata*).

Recombinant protein and inhibitor treatments were conducted as previously described [11], [27]–[29], [32]: bFGF (100 ng/ml) from the 16-cell stage, the protein synthesis inhibitor puromycin (200 µg/ml) from the 8-cell stage, the MEK inhibitor U0126 (4 µM) from the 8-cell stage and the TGFβ type 1 receptor inhibitor SB431542 (5 to 10 µM) from the 16-cell stage.

Standard control-MO (5'-CCTCTTACCTCAGTTACAATTTATA 3') and *otx*-MO (5′-ACATGTTAGGAATTGAACCCGTGGT-3′) were purchased from GeneTools LLC and were injected at 0.25 to 0.50 mM.

### Gene model identifiers

The genes described in this study are represented by the following gene models in the KH2012 *Ciona intestinalis* assembly: *Fgf9/16/20* (KH.C2.125), *Otx* (KH.C4.84), *Nodal* (KH.L106.16), *Msxb* (KH.C2.957), *Delta2* (KH.L50.6), *Chordin* (KH.C6.145), *Klf1/2/4* (KH.C5.154), *KH.C7.391* (KH.C7.391), *Dmrt1* (KH.S544.3), *Lefty* (KH.C3.411), *Fog* (KH.C10.574) and *Ap2-like2* (KH.C7.43).

### *In situ* hybridization, X-gal staining

Whole mount *in situ* hybridization and X-gal staining were performed as previously described [11]. Dig-labeled probes were synthesized from the following cDNAs for *C. intestinalis*: *Msxb* (cign067l18), *Delta2* (cieg005o22), *Chordin* (cign055j01), *Nodal* (cicl090l02), *Dmrt1* (ciad017d15), *Klf1/2/4* (citb012d14), *KH.C7.391* (cilv038e26) [69], *Otx* [27] and *Ap2-like2* (cien223529) (Rothbächer et al., in preparation). For *P. mammillata*: *Msxb* (AHC0AAA214YL10RM1) and *Delta2* (AHC0AAA62YG24RM1). While *Msxb*, *Delta2* and *Chordin* expression in the b6.5 lineage starts at the 64-cell stage (st. 8), we analyzed early gastrula stages (st. 10/11) because expression is much stronger and more readily detectable by *in situ* hybridization.

### Generation of electroporation constructs

Electroporation constructs for overexpression were generated using Gateway technology [51] with the promoter of *Fiend of Gata* (*Fog*) driving expression throughout ectoderm from the 16-cell stage [32], [39]. Constructs for *Fgf9/16/20*, *Nodal* and *Lefty* have already been described [32]. pFOG-Otx was generated by U. Rothbächer using the pENTRY clone cien28442 (Rothbächer et al., in preparation). A construct corresponding to the homeodomain of Otx fused to the Engrailed repressor domain has already been used [42] and was converted into a pENTRY clone using the following primers: attB1-OTXHD-Fw (5′-AAAAAGCAGGCTCAGAAAAAATGGTATACAGTTCGTCTAGAAAAC-3′) and attB2-EnR-Rev (5′-AGAAAGCTGGGTGAATTCTATACGTTCAGGTCCT-3′).

For transcriptional assay, genomic fragments were PCR amplified from sperm genomic DNA using AccuPrime Taq HiFi DNA polymerase (Invitrogen) and converted into pENTRY clones by a BP clonase reaction or TA cloning using the PCR8/GW/TOPO TA cloning kit (Invitrogen). The LR clonase reaction was performed to produce an expression clone with the genomic region in front of the minimal promoter of *Fog* and of nls-LacZ [51]. A detailed list of primers and vectors is described in Table S3. Enhancers msxb-A to -M (Figures 6, S7 and S8) were designed based on the msxb-OtxUP type B sequence and were synthesized as G-blocks Gene Fragments (Integrated DNA Technologies) flanked with AttB sequences (sequences listed in File S1). G-Blocks were shuffled into pDONR221 through BP reaction and through LR reaction into Rfa-bpFOG-nlsLacZ [51].

## Supporting Information

Figure S1 FGF and Nodal signaling disruption effects on neural b-line and epidermis markers expression. Treatment from the 8-cell stage with the MEK inhibitor U0126 led to a loss of *Delta2* (E) and *Chordin* (F) expression in the ectoderm at early gastrula stages (st. 10). *Ap2-like2*, normally expressed in epidermis expression (C, D) is ectopically expressed in a- and b-line neural precursors (white arrowheads) (G, H). *Delta2* is ectopically expressed in vegetal cells. This expression corresponds to an expansion of trunk lateral cell fate (A7.6) where *Delta2* is expressed at the expense of anterior endoderm (A7.5) as previously described [72]. Treatment from the 16-cell stage with the Nodal receptor inhibitor SB431542 also abolished the expression of *Delta2* (I) and *Chordin* (J) in the ectoderm, but the expression of *Ap2-like2* was not modified (K, L). Expression of *Delta2* and *Chordin* in other territories such as lateral A-line neural precursors was also dependent on Nodal as previously reported [29], [35], [73]. Black arrowheads indicate b-line neural precursors. Vegetal views with anterior to the top (A, B, E, F, I and J). Animal view with anterior to the top (C, G and K). Lateral view with anterior to the top (D, H and L). For each panel a schematic animal view (A-C, E-G and I-K) or lateral view (D, H and L) of stage 10 embryo depicts vegetal cells in grey, anterior ectoderm in white, posterior ectoderm in yellow and gene expression in blue.
(PDF)

Figure S2 Direct activation of *Otx* and *Nodal* by FGF signaling independently of Nodal signaling at the 32-cell stage. *Otx* (A) is expressed in the a6.5 and b6.5 blastomeres (neural precursors) at the 32-cell stage (and vegetal blastomeres B6.4), while *Nodal* (B) is only expressed in the b6.5 blastomeres. bFGF treatment from the 16-cell stage led to ectopic activation of *Otx* in all ectodermal cells (C) and to ectopic activation of *Nodal* in all posterior (b-line) ectodermal cells (D). This effect was not modified by co-treatment with the Nodal signaling inhibitor SB431542 (E and F). Activation of *Otx* and *Nodal* by bFGF treatment was not suppressed by prior treatment (from the 8-cell stage) with the protein synthesis inhibitor puromycin (I, J), suggesting direct transcriptional activation. Following treatment with puromycin alone, *Otx* (G) and *Nodal* (H) were not expressed. Activation of *Nodal* expression in the presence of puromycin was detected throughout ectoderm (J) possibly because of inhibition of the anterior determinant FoxA-a [40], [41] by puromycin. Animal views with anterior to the top. For each panel a schematic animal view of 32-cell stage (stage 6) embryos depicts anterior ectoderm in white, posterior ectoderm in yellow and gene expression in blue.
(PDF)

Figure S3 Interactions between *Otx* and *Nodal*. A) *Nodal* expression is dependent on *Otx* and itself, but *Otx* expression is not. Control embryos at the 64-cell stage probed for *Nodal* (i) and *Otx* (v) expression. ii) Overexpression of Otx in the ectoderm *via* the pFOG promoter through electroporation activated *Nodal* expression in a clonal manner. Overexpression of OtxHDenR (iii) or Lefty (iv) repressed *Nodal* expression (white arrows mark repressed expression). *Otx* expression at the 64-cell stage was unaffected by overexpression of either Nodal (vi) or Lefty (vii). B) Overexpression of Lefty does not block Otx mediated activation of b-line neural markers. Control embryos at early gastrula stages probed for *Msxb* (i) and *Delta2* (v) expression. Otx overexpression led to ectopic activation of *Msxb* (iii) and *Delta2* (vii). While Lefty overexpression suppressed *Msxb* (ii) and *Delta2* (vi) expression, it was not sufficient to block the action of Otx though it seemed to reduce the levels of ectopic activation (iv, viii). C) Nodal activation of b-line neural markers in a-line precursors requires Otx. Upon Nodal overexpression, *Msxb* and *Delta2* are ectopically expressed in anterior neural precursors (circled in red). The number of cells with ectopic staining in this territory was determined for every embryo. The graph represents the proportion of embryos with the number of ectopic cells indicated in the key following overexpression of Nodal alone or in combination with OtxHDenR. At the top of each column the mean cell number is indicated. The effect is not massive probably because of the mosaicism observed following electroporation: Nodal can exert its effect on cells that have not received the pFOG-OtxHDenR construct. Animal views with anterior to the top, except in (C) that shows neural plate views.
(PDF)

Figure S4 Overexpression of a dominant negative form of Otx suppresses b6.5 fate. Control embryos probes for *Msxb* (A), *Delta2* (C) and *Ap2-like2* (E, G) at early gastrula stages, and *Klf1/2/4* (I) and *KH.C7.391* (K) at tailbud stages. OtxHDenR [42] overexpression throughout ectoderm using the pFOG driver led to the repression of *Msxb* (B), *Delta2* (D), *Klf1/2/4* (J) and *KH.C7.391* (L) (black arrows). Expression of the epidermis marker *Ap2-like2* was not modified (F, H). Following electroporation, DNA inheritance is mosaic and the resulting phenotypic effects are also mosaic. Animal view with anterior to the top (A-F). Lateral view with anterior to the top (G, H). Lateral view with anterior to the left, dorsal to the top (I-L).
(PDF)

Figure S5 Alignment of the genomic region “Ci-msxb-b6.5 line”. Alignment of “Ci-msxb-b6.5 line” sequences from *C. intestinalis* type A and type B, and *C. savignyi*. Putative transcription factor binding sites are colored: canonical Fox (AAACA) in blue, canonical Otx (GATTA) in red, non-canonical Otx (GHATTA) in orange and SBE (AGAC) in yellow.
(PDF)

Figure S6 Alignment of the genomic region “Ci-delta2-b6.5 line”. Alignment of “Ci-delta2-b6.5 line” sequences from *C. intestinalis* type A and type B, and *C. savignyi*. Putative transcription factor binding sites are colored: canonical Fox (AAACA) in blue, canonical Otx (GATTA) in red, non-canonical Otx (GHATTA) in orange and SBE (AGAC) in yellow.
(PDF)

Figure S7 Deletion analysis of the “Ci-msxb-b6.5 line” enhancer. We generated three additional constructs active in the b6.5 lineage but with variable strengths. The smallest active construct tested (Ci-msxb-B) is 273 bp long and contains 3 Otx, 2 overlapping Fox binding sites and 4 SBEs. Transcriptional activity of the different enhancers was measured as the percentage of embryos with staining in the b6.5 derivatives at late gastrula stages (stage 14). The number of analyzed embryos is listed in Table S1.
(PDF)

Figure S8 Mutational analysis of the msxb-B enhancer. A) Alignment of msxb-B sequences from *C. intestinalis* type A, *C. intestinalis* type B and *C. savignyi*. Putative transcription factor binding sites are boxed and colored: canonical Fox (AAACA) in dark blue, non-canonical Fox (AACA) in light blue, canonical Otx (GATTA) in red, non-canonical Otx (GAATTA) in orange and SBE (AGAC) in yellow. B) Schematic view of Ci-msxb-B enhancer and its mutated versions. Position and orientation of putative transcription factor binding sites are represented by colored arrows with the same color code as in (A). Mutations are depicted by stars. Transcriptional activity of the different enhancers was measured as the percentage of embryos with staining in the b6.5 derivatives at late gastrula stages (stage 14). The number of analyzed embryos is listed in Table S1. None of the constructs led to ectopic staining. C) Sequences of the different sites. Mutated bases are in lower case.
(PDF)

Figure S9 *Msxb* and *Delta2* are expressed in b-line neural precursors in *Phallusia mammillata*. *In situ* hybridization for *Msxb* (A-H) and *Delta2* (J-Q) at the 8-cell (A, J), 32-cell (B, K), 64-cell (C, D, L, M), 92-cell (E, F, N, O) and 112-cell stage (G, H, P, Q). Expression of both genes is virtually identical to what is observed in *Ciona intestinalis*: onset at the 64-cell stage in b7.9 and b7.10 blastomere pairs. *Msxb* is maintained in the daughter cells while *Delta2* is restricted to dorsal tail epidermis midline precursors (b8.18 and b8.20). *Delta2* is also expressed in A7.6 and its daughter cells (A8.11 and A8.12) that are visible through transparency (Q) and A8.15 and A8.16. *Delta2* is detected in a-line neural precursors (a8.25 and a8.26) at early gastrula (112-cell stage) while, in *C. intestinalis*, expression in this territory is not observed before late gastrula stages. A schematic depicts *Msxb* (I) and *Delta2* (R) expression in blue. Lateral views with anterior to the left and animal to the top (A, D, F, H, J, M, O, Q). Animal view with anterior to the left (B, C, E, G). Vegetal view with anterior to the left (K, L, N, P).
(PDF)

Figure S10 The “Pm-msxb-b6.5 line” enhancer. A) Schematic representation of transcription factor binding site composition. B) Genomic browser view of *Pm-Msxb* locus with tested enhancer (grey bar), genomic alignment profiles of *P. mammillata versus P. fumigata*, and *P. mammillata versus C. intestinalis* genomic sequences (black), ESTs contigs (orange) and *ab initio* gene models (green) (extracted from the Aniseed genome browser). C) Alignment of “Pm-msxb-b6.5 line” sequences from *P. mammillata* reference genome, cloned region and *P. fumigata* reference genome. The same color code as in figures 4 and 5 is used: canonical Fox (AAACA) in blue, canonical Otx (GATTA) in red, non-canonical Otx (GHATTA) in orange and SBE (AGAC) in yellow.
(PDF)

Figure S11 The “Ci-msxb-b6.5 line” enhancer is active in anterior neural tissue in both *C. intestinalis* and *P. mammillata* embryos. The “Ci-msxb-b6.5 line” enhancer was electroporated and X-gal staining was performed at late tailbud stages in *C. intestinalis* (A) and *P. mammillata* (B) embryos. Black arrows points to staining in anterior sensory vesicle and anterior neural boundary [42].
(PDF)

Table S1 Transcriptional activity of enhancers tested in *C. intestinalis* embryos.
(PDF)

Table S2 Transcriptional activity of enhancers tested in *P. mammillata* embryos.
(PDF)

Table S3 Primers and vectors used in transcriptional assays.
(PDF)

File S1 Sequences of msxb-B enhancer and its mutated versions.
(PDF)
