# Supplementary figures and images for: An Otx/Nodal Regulatory Signature for Posterior Neural Development in Ascidians
Source: PLoS Genet. 2014 Aug 14;10(8):e1004548. doi: 10.1371/journal.pgen.1004548 (PMC4133040; doi:10.1371/journal.pgen.1004548)

*Delta2**Chordin**Ap2-like2*

DMSO

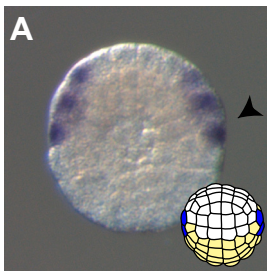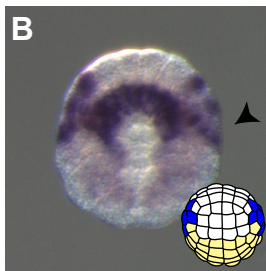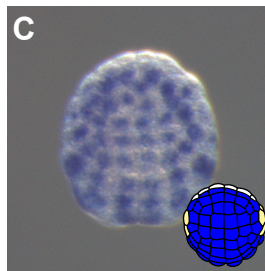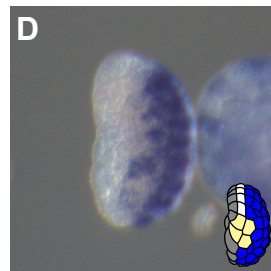

U0126

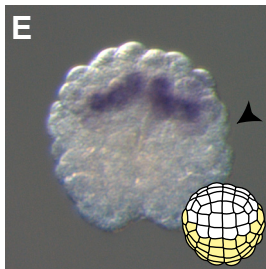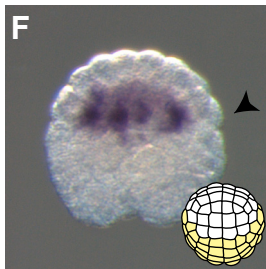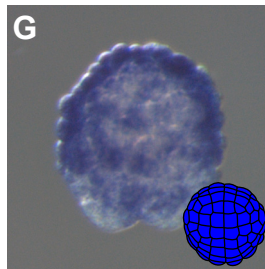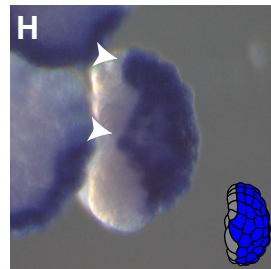

SB431542

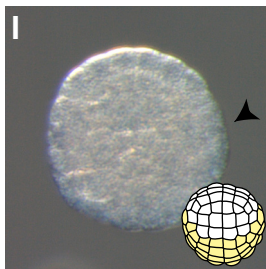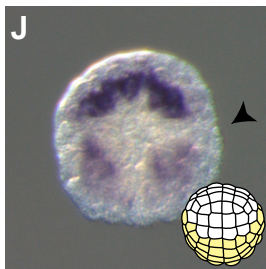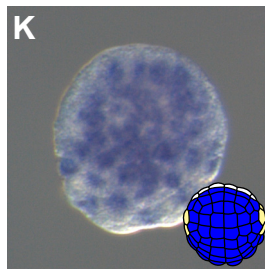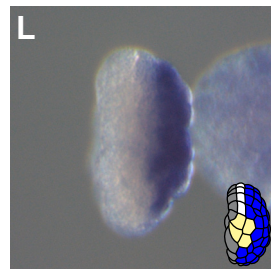

Supplement: Figure S1 — FGF and Nodal signaling disruption effects on neural b-line and epidermis markers expression. Treatment from the 8-cell stage with the MEK inhibitor U0126 led to a loss of Delta2 (E) and Chordin (F) expression in the ectoderm at early gastrula stages (st. 10). Ap2-like2, normally expressed in epidermis expression (C, D) is ectopically expressed in a- and b-line neural precursors (white arrowheads) (G, H). Delta2 is ectopically expressed in vegetal cells. This expression corresponds to an expansion of trunk lateral cell fate (A7.6) where Delta2 is expressed at the expense of anterior endoderm (A7.5) as previously described [72]. Treatment from the 16-cell stage with the Nodal receptor inhibitor SB431542 also abolished the expression of Delta2 (I) and Chordin (J) in the ectoderm, but the expression of Ap2-like2 was not modified (K, L). Expression of Delta2 and Chordin in other territories such as lateral A-line neural precursors was also dependent on Nodal as previously reported [29], [35], [73]. Black arrowheads indicate b-line neural precursors. Vegetal views with anterior to the top (A, B, E, F, I and J). Animal view with anterior to the top (C, G and K). Lateral view with anterior to the top (D, H and L). For each panel a schematic animal view (A-C, E-G and I-K) or lateral view (D, H and L) of stage 10 embryo depicts vegetal cells in grey, anterior ectoderm in white, posterior ectoderm in yellow and gene expression in blue. (PDF) [file pgen.1004548.s001.pdf]

*Otx**Nodal*

control

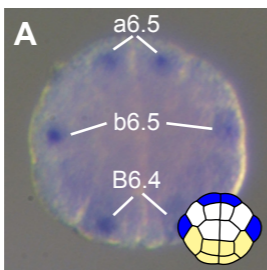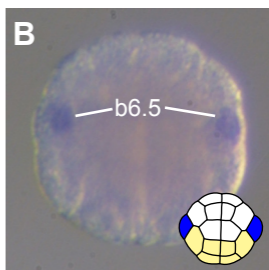

bFGF

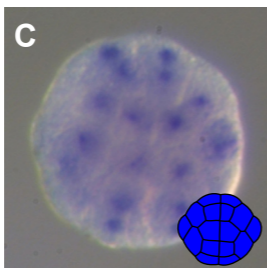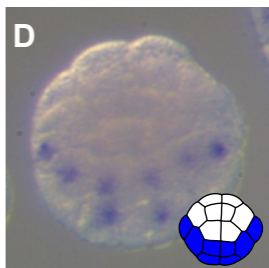bFGF+  
SB431542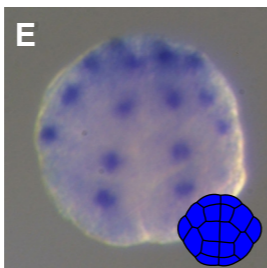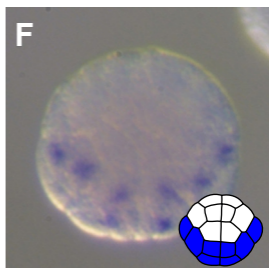

puromycin

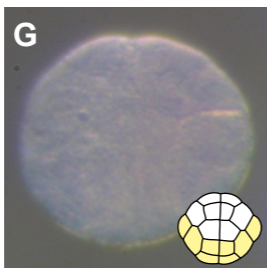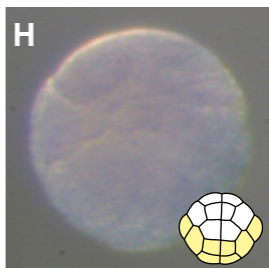bFGF+  
puromycin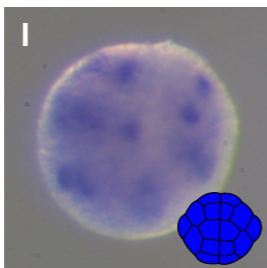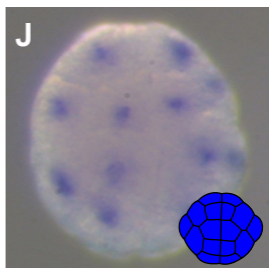

Supplement: Figure S2 — Direct activation of Otx and Nodal by FGF signaling independently of Nodal signaling at the 32-cell stage. Otx (A) is expressed in the a6.5 and b6.5 blastomeres (neural precursors) at the 32-cell stage (and vegetal blastomeres B6.4), while Nodal (B) is only expressed in the b6.5 blastomeres. bFGF treatment from the 16-cell stage led to ectopic activation of Otx in all ectodermal cells (C) and to ectopic activation of Nodal in all posterior (b-line) ectodermal cells (D). This effect was not modified by co-treatment with the Nodal signaling inhibitor SB431542 (E and F). Activation of Otx and Nodal by bFGF treatment was not suppressed by prior treatment (from the 8-cell stage) with the protein synthesis inhibitor puromycin (I, J), suggesting direct transcriptional activation. Following treatment with puromycin alone, Otx (G) and Nodal (H) were not expressed. Activation of Nodal expression in the presence of puromycin was detected throughout ectoderm (J) possibly because of inhibition of the anterior determinant FoxA-a [40], [41] by puromycin. Animal views with anterior to the top. For each panel a schematic animal view of 32-cell stage (stage 6) embryos depicts anterior ectoderm in white, posterior ectoderm in yellow and gene expression in blue. (PDF) [file pgen.1004548.s002.pdf]

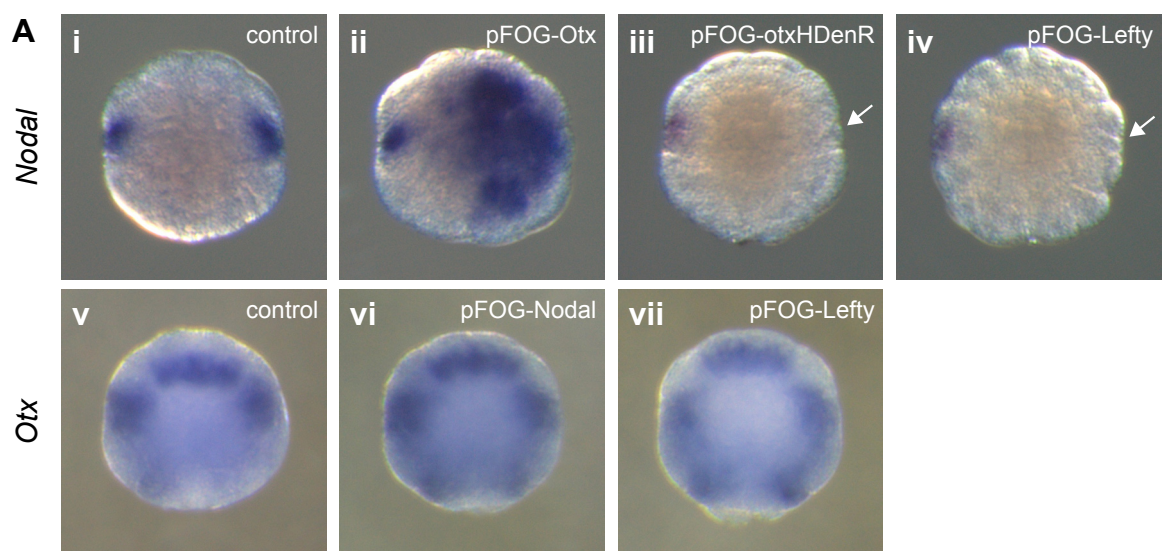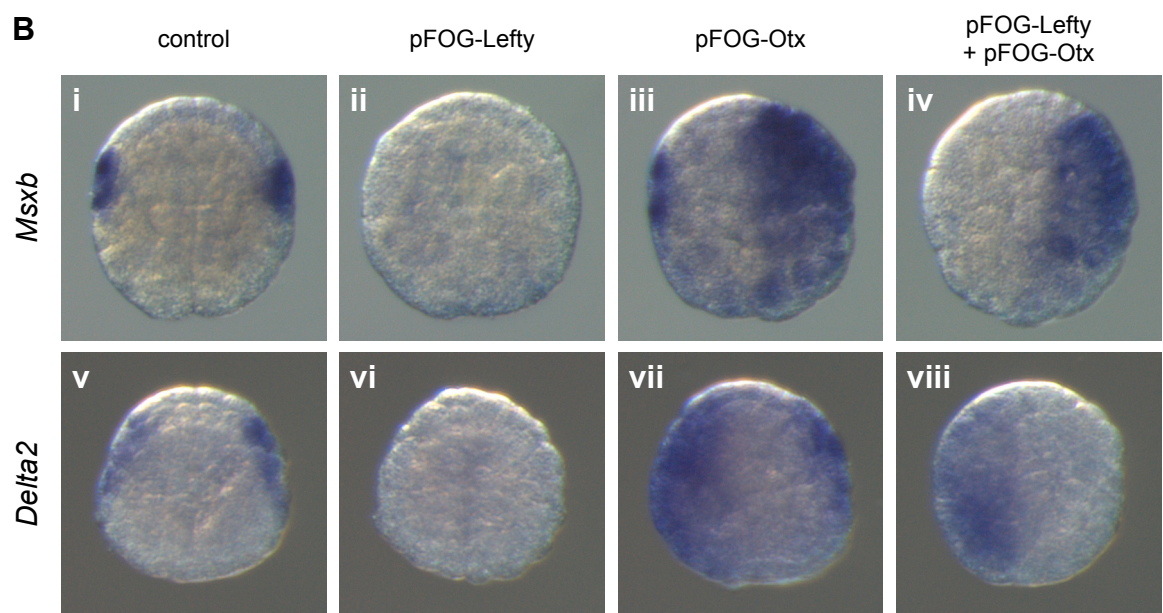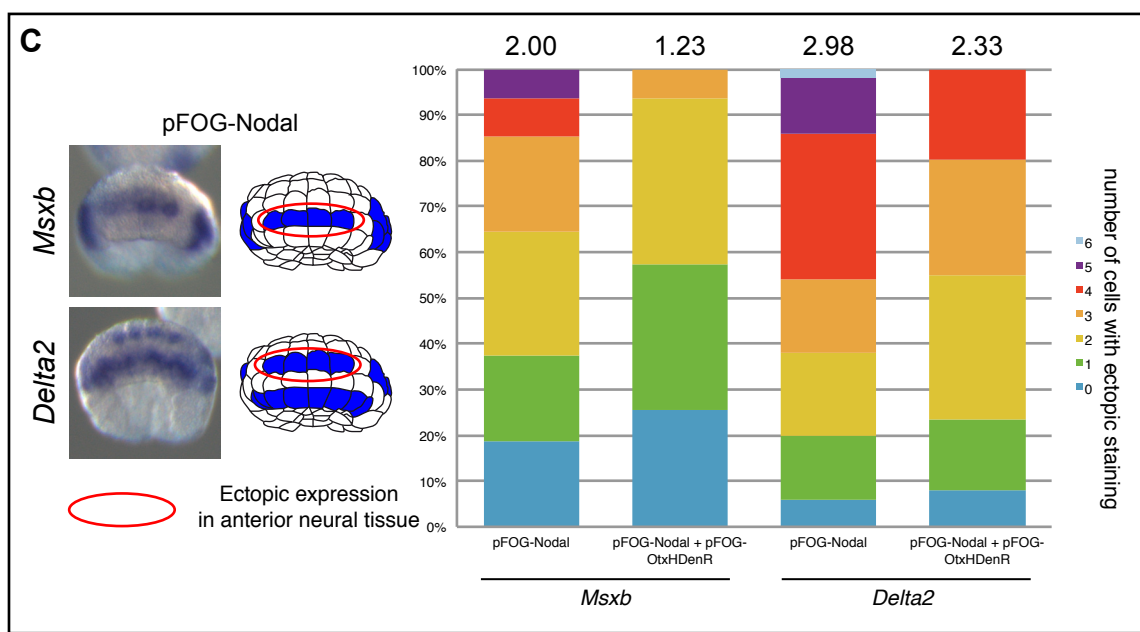

Supplement: Figure S3 — Interactions between Otx and Nodal. A) Nodal expression is dependent on Otx and itself, but Otx expression is not. Control embryos at the 64-cell stage probed for Nodal (i) and Otx (v) expression. ii) Overexpression of Otx in the ectoderm via the pFOG promoter through electroporation activated Nodal expression in a clonal manner. Overexpression of OtxHDenR (iii) or Lefty (iv) repressed Nodal expression (white arrows mark repressed expression). Otx expression at the 64-cell stage was unaffected by overexpression of either Nodal (vi) or Lefty (vii). B) Overexpression of Lefty does not block Otx mediated activation of b-line neural markers. Control embryos at early gastrula stages probed for Msxb (i) and Delta2 (v) expression. Otx overexpression led to ectopic activation of Msxb (iii) and Delta2 (vii). While Lefty overexpression suppressed Msxb (ii) and Delta2 (vi) expression, it was not sufficient to block the action of Otx though it seemed to reduce the levels of ectopic activation (iv, viii). C) Nodal activation of b-line neural markers in a-line precursors requires Otx. Upon Nodal overexpression, Msxb and Delta2 are ectopically expressed in anterior neural precursors (circled in red). The number of cells with ectopic staining in this territory was determined for every embryo. The graph represents the proportion of embryos with the number of ectopic cells indicated in the key following overexpression of Nodal alone or in combination with OtxHDenR. At the top of each column the mean cell number is indicated. The effect is not massive probably because of the mosaicism observed following electroporation: Nodal can exert its effect on cells that have not received the pFOG-OtxHDenR construct. Animal views with anterior to the top, except in (C) that shows neural plate views. (PDF) [file pgen.1004548.s003.pdf]

control

pFOG-otxHDenR

A

B

*Msxb*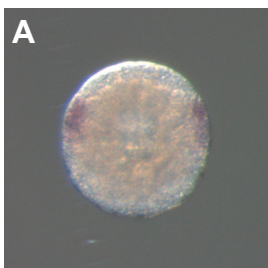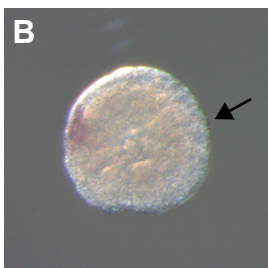

C

D

*Delta2*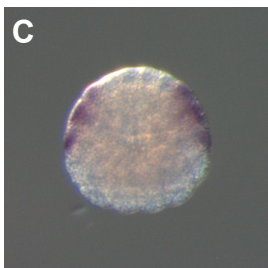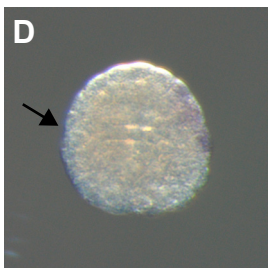

E

F

*Ap2-like2*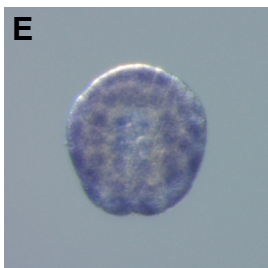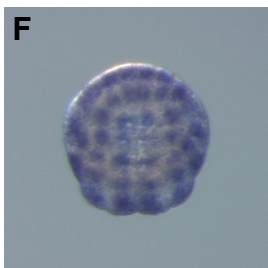

G

H

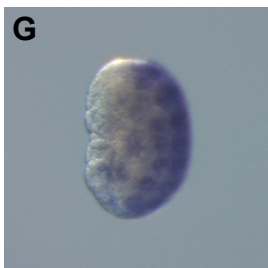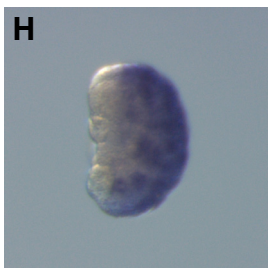

I

J

*Klf1/2/4*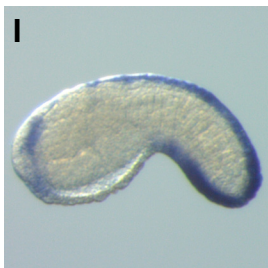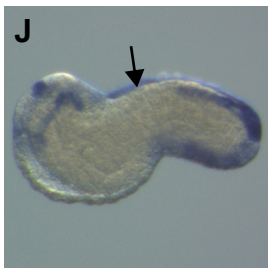

K

L

*KH.C7.391*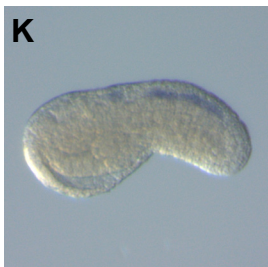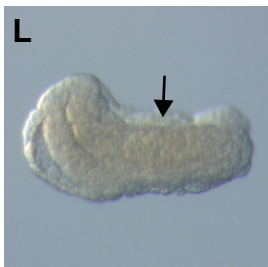

Supplement: Figure S4 — Overexpression of a dominant negative form of Otx suppresses b6.5 fate. Control embryos probes for Msxb (A), Delta2 (C) and Ap2-like2 (E, G) at early gastrula stages, and Klf1/2/4 (I) and KH.C7.391 (K) at tailbud stages. OtxHDenR [42] overexpression throughout ectoderm using the pFOG driver led to the repression of Msxb (B), Delta2 (D), Klf1/2/4 (J) and KH.C7.391 (L) (black arrows). Expression of the epidermis marker Ap2-like2 was not modified (F, H). Following electroporation, DNA inheritance is mosaic and the resulting phenotypic effects are also mosaic. Animal view with anterior to the top (A-F). Lateral view with anterior to the top (G, H). Lateral view with anterior to the left, dorsal to the top (I-L). (PDF) [file pgen.1004548.s004.pdf]

Activity

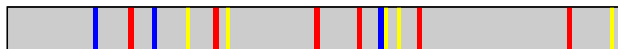

Ci-msxb-b6.5 line (707 bp)

78%

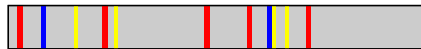

Ci-msxb-OtxUP (478 bp)

34%

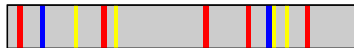

Ci-msxb-A (402 bp)

60%

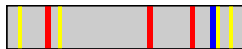

Ci-msxb-B (273 bp)

55%

Otx SBE Fox

Supplement: Figure S7 — Deletion analysis of the “Ci-msxb-b6.5 line” enhancer. We generated three additional constructs active in the b6.5 lineage but with variable strengths. The smallest active construct tested (Ci-msxb-B) is 273 bp long and contains 3 Otx, 2 overlapping Fox binding sites and 4 SBEs. Transcriptional activity of the different enhancers was measured as the percentage of embryos with staining in the b6.5 derivatives at late gastrula stages (stage 14). The number of analyzed embryos is listed in Table S1. (PDF) [file pgen.1004548.s007.pdf]

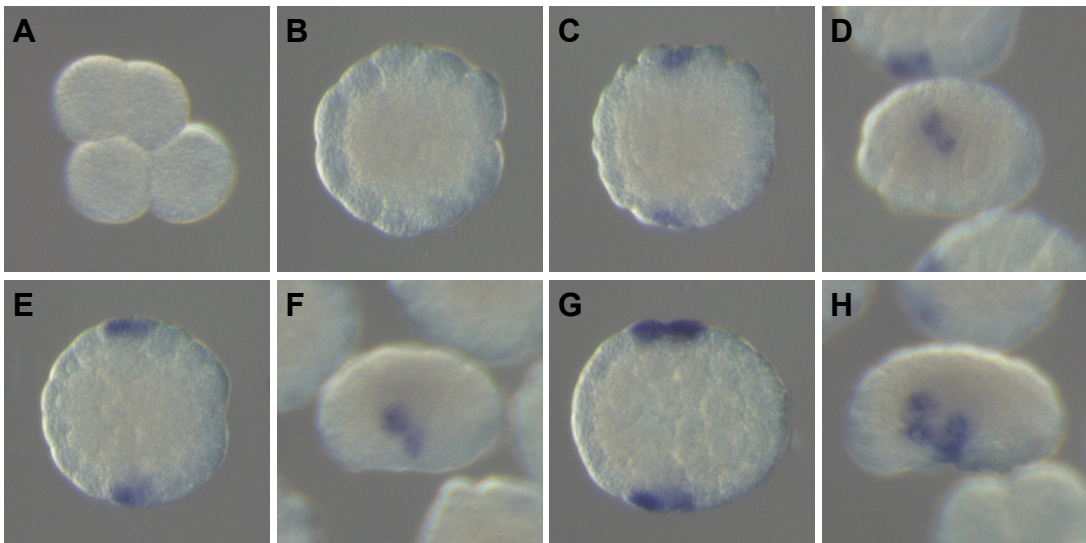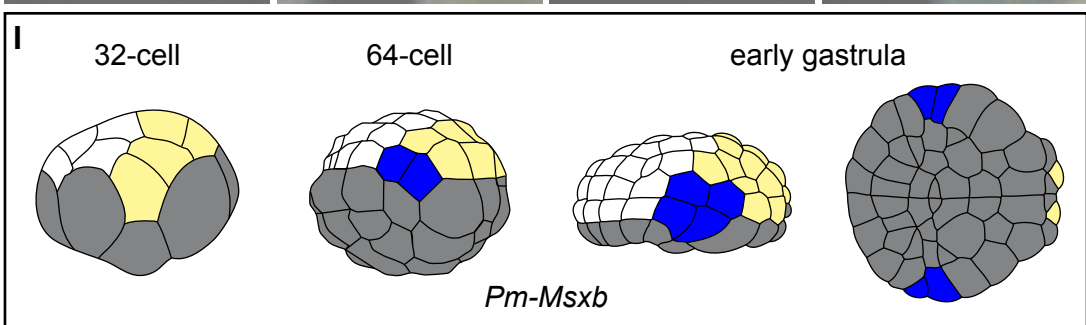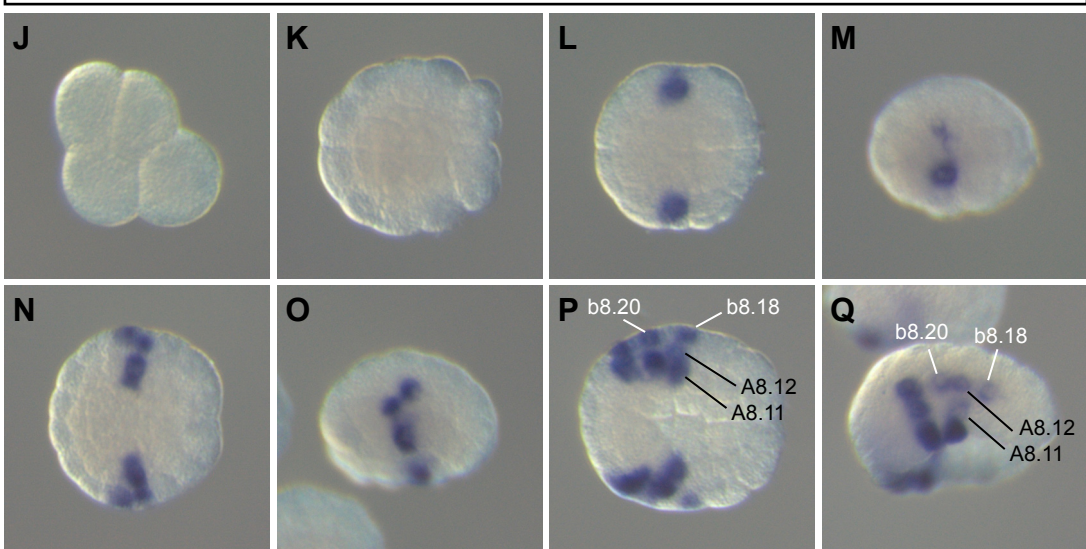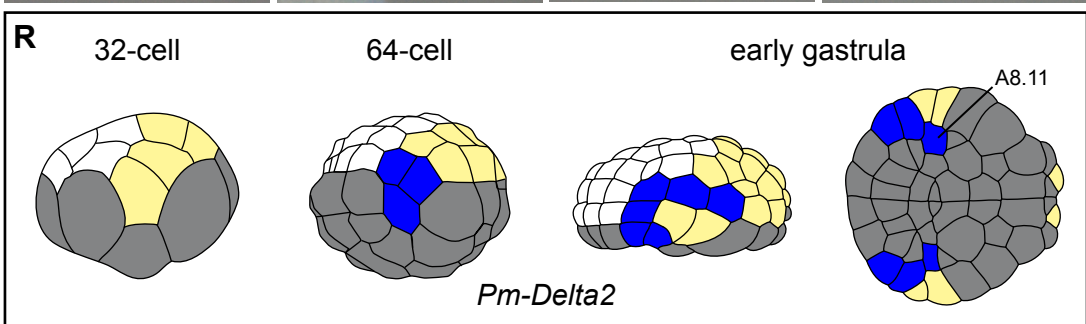

Supplement: Figure S9 — Msxb and Delta2 are expressed in b-line neural precursors in Phallusia mammillata. In situ hybridization for Msxb (A-H) and Delta2 (J-Q) at the 8-cell (A, J), 32-cell (B, K), 64-cell (C, D, L, M), 92-cell (E, F, N, O) and 112-cell stage (G, H, P, Q). Expression of both genes is virtually identical to what is observed in Ciona intestinalis: onset at the 64-cell stage in b7.9 and b7.10 blastomere pairs. Msxb is maintained in the daughter cells while Delta2 is restricted to dorsal tail epidermis midline precursors (b8.18 and b8.20). Delta2 is also expressed in A7.6 and its daughter cells (A8.11 and A8.12) that are visible through transparency (Q) and A8.15 and A8.16. Delta2 is detected in a-line neural precursors (a8.25 and a8.26) at early gastrula (112-cell stage) while, in C. intestinalis, expression in this territory is not observed before late gastrula stages. A schematic depicts Msxb (I) and Delta2 (R) expression in blue. Lateral views with anterior to the left and animal to the top (A, D, F, H, J, M, O, Q). Animal view with anterior to the left (B, C, E, G). Vegetal view with anterior to the left (K, L, N, P). (PDF) [file pgen.1004548.s009.pdf]

Recipient embryo species

*Ciona intestinalis*

*Phallusia mammillata*

Ci-msxb-b6.5 line enhancer

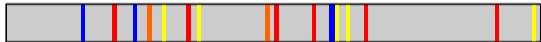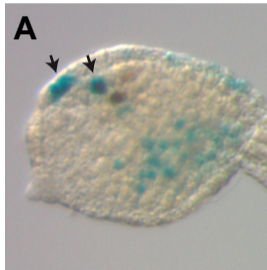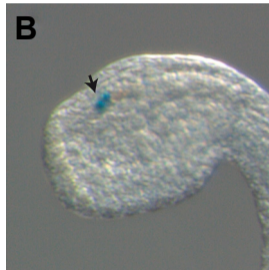

Supplement: Figure S11 — The “Ci-msxb-b6.5 line” enhancer is active in anterior neural tissue in both C. intestinalis and P. mammillata embryos. The “Ci-msxb-b6.5 line” enhancer was electroporated and X-gal staining was performed at late tailbud stages in C. intestinalis (A) and P. mammillata (B) embryos. Black arrows points to staining in anterior sensory vesicle and anterior neural boundary [42]. (PDF) [file pgen.1004548.s011.pdf]
