# Supplementary material for: An Otx/Nodal Regulatory Signature for Posterior Neural Development in Ascidians
Source: PLoS Genet. 2014 Aug 14;10(8):e1004548. doi: 10.1371/journal.pgen.1004548 (PMC4133040; doi:10.1371/journal.pgen.1004548)
Supplement: Figure S5 — Alignment of the genomic region “Ci-msxb-b6.5 line”. Alignment of “Ci-msxb-b6.5 line” sequences from C. intestinalis type A and type B, and C. savignyi. Putative transcription factor binding sites are colored: canonical Fox (AAACA) in blue, canonical Otx (GATTA) in red, non-canonical Otx (GHATTA) in orange and SBE (AGAC) in yellow. (PDF) [file pgen.1004548.s005.pdf]

```
Ci-msxb-b6.5 line_typeA      AGCAATCATTTTGAGCTTCCACATTTGATATTAAAATTCTCGTATTTGTTGTTATTTATT
Ci-msxb-b6.5 line_typeB      AGCAATCATTTTGAGCTTCCACATTTGATATTAAAACTCTGATATTTGTTATTTTTTAC-
Cs-msxb-b6.5 line            AATAATTGTTATTAGTGGTGTAGTTTTAGGTTAAC-TAATTTTATTTGACCTCTAATGTA
                             *  ***  ** * **        *** *  ****     *  ******   *    *

Ci-msxb-b6.5 line_typeA      AATGATTTTGAGTGACTTTTTTAGACTCAGTTTTTGTTT-TGTTTTTCAATTATTCAAGT
Ci-msxb-b6.5 line_typeB      AATGGTTTTTGGTGACTTTTTGGACTTTTAGTTAAATTTGTGTTTTTCAATTATTCAAGT
Cs-msxb-b6.5 line            TATTATTTT-----ACCATTTAAAAATGAAACCGACTTT----------ATTAGGCCAAA
                              **  ****     **  ***     *         ***          ****  * *

Ci-msxb-b6.5 line_typeA      TCGGATGTGTGGAGATA--CGGGATTAATACAATGAATGTTCAATTAAAA-ACACAAAAG
Ci-msxb-b6.5 line_typeB      TCGGATGTGTGGAGATATACGGGATTAATATAATGATTGTTCAATTAAAA-ACACAAAAG
Cs-msxb-b6.5 line            G-GGATGGTAAAGGATATATGGT-TTATTATAATCATTGTTCAATTTAATTAAACAAAAG
                               *****      ****   **  *** ** *** * ********* **  * *******

Ci-msxb-b6.5 line_typeA      TTTTAATTTGAATTAATGTCGGCGCTGCAGACGAGCCGAGAGCTTGTCGGATTTAAAATG
Ci-msxb-b6.5 line_typeB      TTTTAATTTGAATTAATGTCGGCGCTGCAGACGAGCCGAGAGCTTGTCGGATTTAAAATG
Cs-msxb-b6.5 line            TT-------GAATTGTTGTTATTGCTGCAGACGA--CAATAGTAGATCGGATTTTAATTT
                             **       *****  ***    ***********  * * **    ******** ** *

Ci-msxb-b6.5 line_typeA      TAATCCTCCAA--TTTTGTCTGGCACTGACGGTGTAGATGCAATCTGAAAATGGCGACTG
Ci-msxb-b6.5 line_typeB      TAATCCTCTAA--CTTTGTCTGGCACTGACGGTGTAGATGCAATCTGAAAATGGCGGCTG
Cs-msxb-b6.5 line            TAATCCGCAAAACTATAGTCTACGACTGA------AGAGCATATCTTAAAATGGCGGCCA
                             ****** * **    * ****   *****      ***    **** ********* *

Ci-msxb-b6.5 line_typeA      AGCAGGATCGGGTCGCGGCTGGAGCTCCGGCGGGCTCGGGACGCTCATAATTCCGCCGGT
Ci-msxb-b6.5 line_typeB      AGCAGGATCGGGTCGCGGCTGGAGCTCCGGCGGGCTCGGGACGCTCATAATTCCGCCGGT
Cs-msxb-b6.5 line            AGAAGGATCGACGCGCGGCTGGATCTCCGACGGGCTCGGGACGCTCGTAATCCGGCCTAT
                             ** *******   ********** ***** **************** **** * ***  *

Ci-msxb-b6.5 line_typeA      AATCCCC-GTAACGTCGA------------TGAAAGCGAACGCGCCGACAAAAGTGACGA
Ci-msxb-b6.5 line_typeB      AATCCCC-GTAACGTCGA------------TGAAAGCGAACGCGCCGACAAAAGTGACGA
Cs-msxb-b6.5 line            AATCCCCCGCAACGTCGACCATTCGACGGATGAAAGGGGAGGTACCGACAAAAGTGACAA
                             ******* * ********            ****** * * *  ************** *

Ci-msxb-b6.5 line_typeA      AGATTAAGTGTAACAACATGGTTTAAACAAACAGACTGGAGCAGCGGAGACGAGAGAGAG
Ci-msxb-b6.5 line_typeB      AGATTAAGTGTAACAACATGGTTTAAACAAACAGACTGGAGCAGCGGAGACGAGAGAGAG
Cs-msxb-b6.5 line            AGATTAAGTGTAGCAACAAGCTTTAAACAAACAGACTGGAGAGAGAAACGAGACAACGCG
                             ************ ***** * ********************      *   ** *  * *

Ci-msxb-b6.5 line_typeA      AG---GGAACGTA--ATCGCCGCGGCCGAAGCGTGACCAAGTCGTTGTATTGTTATAATA
Ci-msxb-b6.5 line_typeB      AGA-GGGAGCGTA--ATCGCCGCGGCCGAAGCGTGACCAAGTCGTTGTATTGTTATAATA
Cs-msxb-b6.5 line            AGATAATAGCGTAGAATGGACGAGATGCGAGCGTGACCAACTCGCTGTATTGTTATAATC
                             **     * ****  ** * ** *     *********** *** **************

Ci-msxb-b6.5 line_typeA      GAAAATCGTTTTATTATGATTTAATGAACGCGCGCTGGCGACTGAAGGATGCTGTATAAT
Ci-msxb-b6.5 line_typeB      GAAAATCGTTTTATTATGATTTAACGAACGCGCGCTGGCGACTGAAGGATGCTGTATAAT
Cs-msxb-b6.5 line            GAAAATCGTTTTATTATGATTTAATGAAGGCAGGCTGGCGGCTGAAGGAAGCGAAATAAT
                             ************************ *** **  ******* ******** **   *****

Ci-msxb-b6.5 line_typeA      TGGGGG-TAATGGGGAAATATCGGGAGAAAAAATATGGCGATCT----GCGGGAGAGGAT
Ci-msxb-b6.5 line_typeB      TGGGGG-TAATGGGGAAATATCGGGAGAAAAAATATGGCGATCT----GCGGGAGAGGAT
Cs-msxb-b6.5 line            TGGGGGGTAATGTACGGGTACGGCGAGAAAAAATATCCCGACGTCGATGCGGGAGAATAT
                             ****** *****      **  * ************  ***  *    ********  **

Ci-msxb-b6.5 line_typeA      AGTCGCTTACATAATCTCTGGAGTTTAGTTGAAGCAGTTCATAT-TGAGAGAAGCATCCC
Ci-msxb-b6.5 line_typeB      AGTCGCTTACATAATCTCTGGAGTTCAGTTGAAGCAGTTCATAT-TGAGAGAAGCATCCC
Cs-msxb-b6.5 line            AGCTGCT-ACAAAATCTCTCGT-CTCAGATGGAAAAGTTTATTTACGATCCAGTCGTCCA
                             **  *** *** ******* *   * ** ** *  **** ** *  **   *  * ***

Ci-msxb-b6.5 line_typeA      TCAGACGAGTAGT
Ci-msxb-b6.5 line_typeB      TCAGACGAGTAGT
Cs-msxb-b6.5 line            AGAGATTTGTGAA
                               ***   **
```
