# Supplementary material for: An Otx/Nodal Regulatory Signature for Posterior Neural Development in Ascidians
Source: PLoS Genet. 2014 Aug 14;10(8):e1004548. doi: 10.1371/journal.pgen.1004548 (PMC4133040; doi:10.1371/journal.pgen.1004548)
Supplement: Figure S6 — Alignment of the genomic region “Ci-delta2-b6.5 line”. Alignment of “Ci-delta2-b6.5 line” sequences from C. intestinalis type A and type B, and C. savignyi. Putative transcription factor binding sites are colored: canonical Fox (AAACA) in blue, canonical Otx (GATTA) in red, non-canonical Otx (GHATTA) in orange and SBE (AGAC) in yellow. (PDF) [file pgen.1004548.s006.pdf]

```
Ci-delta2-b6.5 line_typeA      ACTGTCGCGCTTTATTTTAGTTTGTTTTTAGTCGCGCGCCTTTCTTACTA
Ci-delta2-b6.5 line_typeB      ACTGTCGCGCTTTATTTTAGTTTGTTTTTAGTCGCGCGCCTTTCTTACTA
Cs-delta2-b6.5 line            ACTGTCGCGCTCAGTTTTAGTTTGTTTTTAGTCGCCCGCCTTTCTCTCGA
                               ***********   ********************* *********  * *

Ci-delta2-b6.5 line_typeA      C-AGCTCGTGTTTCGGC-CCCGCTACTAATCGCTGCTGAGTAGTCTGCCA
Ci-delta2-b6.5 line_typeB      C-AGCTCGTGTTACGGC-CTCGCTACTAATCGCTGCTGAGTAGTCTGCCA
Cs-delta2-b6.5 line            CGAGGTCGTGTTACTGCGCTCGTTAGTAATCGCTGCTGAGTAGTCTGCCG
                               * ** ******* * ** * ** ** ***********************

Ci-delta2-b6.5 line_typeA      CTCTGCGCGGGGCAGGTGTTATCGTGGGCAGTGGAATATTCCCTTAAAGA
Ci-delta2-b6.5 line_typeB      CTCCGTGCGGGGCAGGTGTTATCGTGGCCAGTGGAATATTCCCTTAAAGA
Cs-delta2-b6.5 line            CTCATACCAGGGCAGGTGTCAGCGTCGTCTGCCCACTATTGCCTATAAGA
                               ***    * ********** * *** * * *   * **** ***  ****

Ci-delta2-b6.5 line_typeA      GCTGAGTCCCAGTCGCTAGTGGATCATAACACAGTCGGTCCAGTGGC-GT
Ci-delta2-b6.5 line_typeB      GCTGTGTCCCAGTCGCTAGTGGATCATAACACAGTCGGTCCAGTGGC-GA
Cs-delta2-b6.5 line            CCCG-GCACTAGTCGACTTTGGATCATAACACAGTCGTTCGTCAACCAGT
                                * * *  * *****    ****************** **      * *

Ci-delta2-b6.5 line_typeA      GCTCTCTCTATTTTACTTGGCTGCCATGCAAGTGGTAGACGGAAGGATTA
Ci-delta2-b6.5 line_typeB      GCTCTCTCTATTTTACTTGGCTGCCATGCAAGTGTTAGACGGAAGGATTA
Cs-delta2-b6.5 line            GCACTCTCTATTTTCATACGCTACT-TGTAAGTGCCAGACGGAAGGATTA
                               ** ***********  *  *** *  ** *****  **************

Ci-delta2-b6.5 line_typeA      AAGGTTTGTTTGTGTCGAT--CGCTTTTAGCTCAGAGATGCATTGAGTCG
Ci-delta2-b6.5 line_typeB      ACGGTTTGTTTGTGTCGAT--CGCTTCTAGCTCAGAGATGCATTGAATCG
Cs-delta2-b6.5 line            TAGATTCGTTTGCAAGCATTGCGCTTCTAGCTCATAGACGAATAGAGTCG
                                 * ** *****     **  ***** ******* *** * ** ** ***

Ci-delta2-b6.5 line_typeA      TAGACTATAGGAGGATTATTACGAAACAATAGCGGCGCACGACCATGATT
Ci-delta2-b6.5 line_typeB      TAGACTATAGGAGGATTATTACGAAACAATAGCGGCGCGCGACCATGATT
Cs-delta2-b6.5 line            TTGACTGTAGCTGGATTATGACGAAACAATGGGGCCGCAAGAGCAGGATT
                               * **** ***  ******* ********** * * ***  ** ** ****

Ci-delta2-b6.5 line_typeA      ATTACGTCAGGATTAACAATTTAAAACGTCGCAAAAAAATCAGCAAA-
Ci-delta2-b6.5 line_typeB      ATTACGTCAGGATTAACAATTTAAAACGTCGCAAAAAAATCAGCAAA-
Cs-delta2-b6.5 line            ATAGAGGCAGGATTAACAATTTAAAATGTCGTCGAAAAAAGATCAAGA
                               **   * ******************* ****   *****  * ***
```
