# Supplementary material for: An Otx/Nodal Regulatory Signature for Posterior Neural Development in Ascidians
Source: PLoS Genet. 2014 Aug 14;10(8):e1004548. doi: 10.1371/journal.pgen.1004548 (PMC4133040; doi:10.1371/journal.pgen.1004548)
Supplement: Figure S8 — Mutational analysis of the msxb-B enhancer. A) Alignment of msxb-B sequences from C. intestinalis type A, C. intestinalis type B and C. savignyi. Putative transcription factor binding sites are boxed and colored: canonical Fox (AAACA) in dark blue, non-canonical Fox (AACA) in light blue, canonical Otx (GATTA) in red, non-canonical Otx (GAATTA) in orange and SBE (AGAC) in yellow. B) Schematic view of Ci-msxb-B enhancer and its mutated versions. Position and orientation of putative transcription factor binding sites are represented by colored arrows with the same color code as in (A). Mutations are depicted by stars. Transcriptional activity of the different enhancers was measured as the percentage of embryos with staining in the b6.5 derivatives at late gastrula stages (stage 14). The number of analyzed embryos is listed in Table S1. None of the constructs led to ectopic staining. C) Sequences of the different sites. Mutated bases are in lower case. (PDF) [file pgen.1004548.s008.pdf]

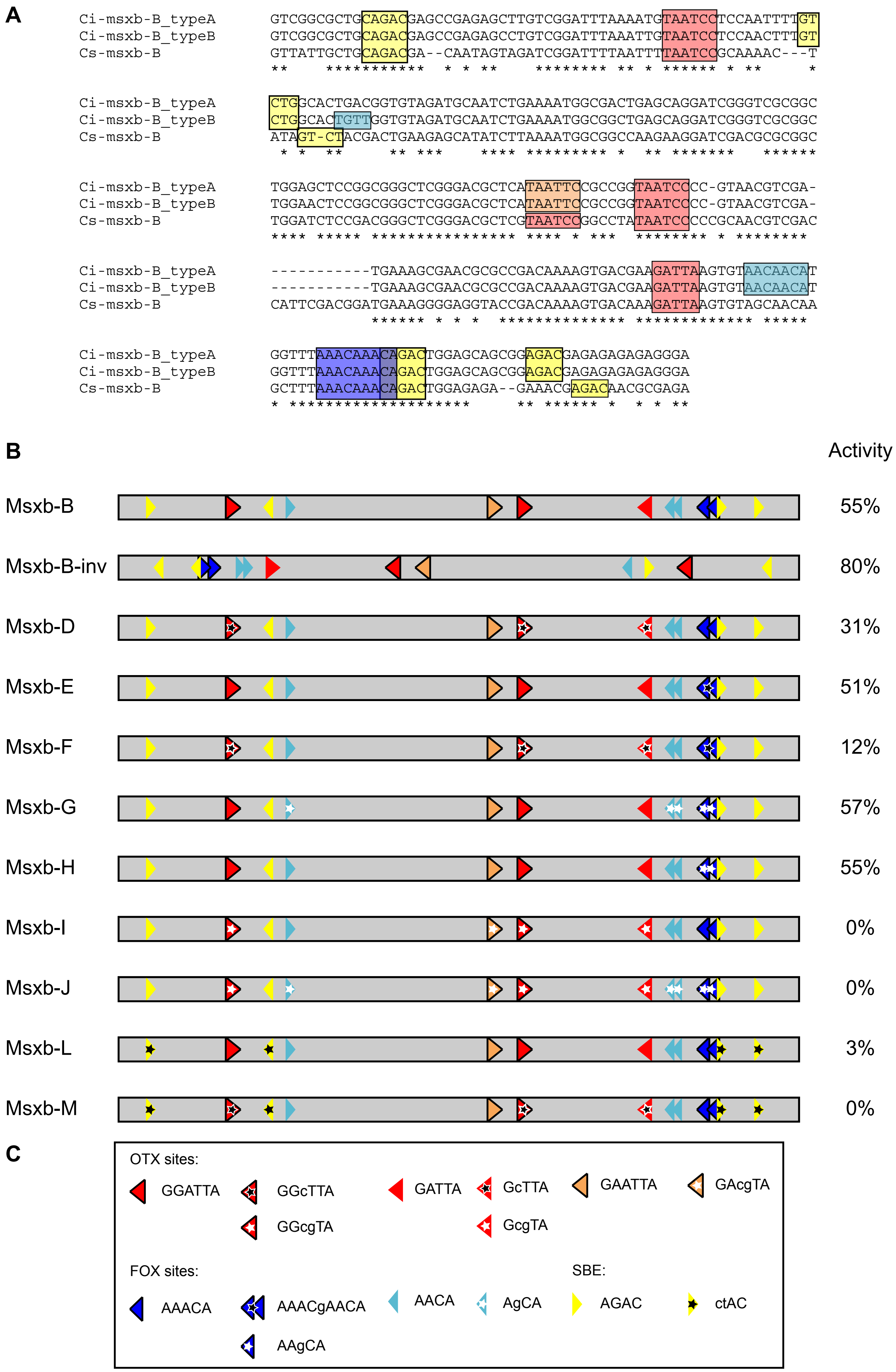

A
Ci-msxb-B_typeA GTCGGCGCTGCAGACGAGCCGAGAGCTTGTCGGATTTAAAATGTAATCCTCCAATTTTGT
Ci-msxb-B_typeB GTCGGCGCTGCAGACGAGCCGAGAGCCTGTCGGATTTAAATTGTAATCCTCCAACTTTGT
Cs-msxb-B GTTATTGCTGCAGACGA--CAATAGTAGATCGGATTTTAATTTTAATCCGCAAAAC---T
Ci-msxb-B_typeA CTGGCACTGACGGTGTAGATGCAATCTGAAAATGGCGACTGAGCAGGATCGGGTCGCGGC
Ci-msxb-B_typeB CTGGCACTGTTGGTGTAGATGCAATCTGAAAATGGCGGCTGAGCAGGATCGGGTCGCGGC
Cs-msxb-B ATAGT-CTACGACTGAAGAGCATATCTTAAAATGGCGGCCAAGAAGGATCGACGCGCGGC
Ci-msxb-B_typeA TGGAGCTCCGGCGGGCTCGGGACGCTCATAATTCCGCCGGTAATCCCC-GTAACGTCGA-
Ci-msxb-B_typeB TGGAACTCCGGCGGGCTCGGGACGCTCATAATTCCGCCGGTAATCCCC-GTAACGTCGA-
Cs-msxb-B TGGATCTCCGACGGGCTCGGGACGCTCGTAATCCGGCCTATAATCCCCCGCAACGTCGAC
Ci-msxb-B_typeA -----------TGAAAGCGAACGCGCCGACAAAAGTGACGAAGATTAAGTGTAACAACAT
Ci-msxb-B_typeB -----------TGAAAGCGAACGCGCCGACAAAAGTGACGAAGATTAAGTGTAACAACAT
Cs-msxb-B CATTCGACGGATGAAAGGGGAGGTACCGACAAAAGTGACAAAGATTAAGTGTAGCAACAA
Ci-msxb-B_typeA GGTTTAAACAAACAGACTGGAGCAGCGGAGACGAGAGAGAGAGGGA
Ci-msxb-B_typeB GGTTTAAACAAACAGACTGGAGCAGCGGAGACGAGAGAGAGAGGGA
Cs-msxb-B GCTTTAAACAAACAGACTGGAGAGA--GAAACGAGACAACGCGAGA
B
Activity
Msxb-B 55%
Msxb-B-inv 80%
Msxb-D 31%
Msxb-E 51%
Msxb-F 12%
Msxb-G 57%
Msxb-H 55%
Msxb-I 0%
Msxb-J 0%
Msxb-L 3%
Msxb-M 0%
C
OTX sites:
GGATTA
GGcTTA
GATTA
GcTTA
GAATTA
GAcgTA
GGcgTA
GcgTA
FOX sites:
AAACA
AAACgAACA
AACA
AgCA
AAgCA
SBE:
AGAC
ctAC
