# Supplementary material for: An Otx/Nodal Regulatory Signature for Posterior Neural Development in Ascidians
Source: PLoS Genet. 2014 Aug 14;10(8):e1004548. doi: 10.1371/journal.pgen.1004548 (PMC4133040; doi:10.1371/journal.pgen.1004548)
Supplement: Figure S10 — The “Pm-msxb-b6.5 line” enhancer. A) Schematic representation of transcription factor binding site composition. B) Genomic browser view of Pm-Msxb locus with tested enhancer (grey bar), genomic alignment profiles of P. mammillata versus P. fumigata, and P. mammillata versus C. intestinalis genomic sequences (black), ESTs contigs (orange) and ab initio gene models (green) (extracted from the Aniseed genome browser). C) Alignment of “Pm-msxb-b6.5 line” sequences from P. mammillata reference genome, cloned region and P. fumigata reference genome. The same color code as in figures 4 and 5 is used: canonical Fox (AAACA) in blue, canonical Otx (GATTA) in red, non-canonical Otx (GHATTA) in orange and SBE (AGAC) in yellow. (PDF) [file pgen.1004548.s010.pdf]

A **B**

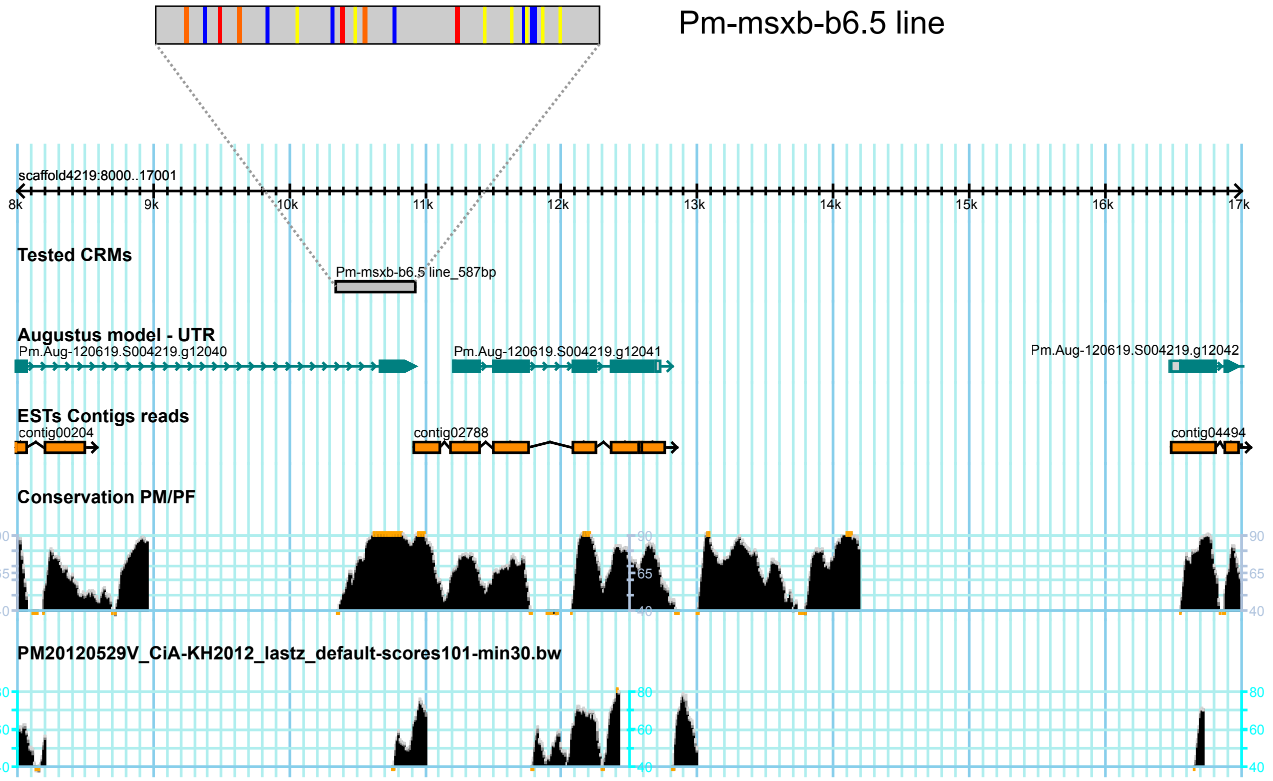

C

```
Pm-msxb-b6.5 line         CTTGTTGAAATAGTTTGAAATCTTCCTGATAATAGT----GCGAATTAAATGACCATTTT
Pm-msxb-b6.5 line_tested  CTTGTTGAAATAGTTTGAAATCTTCCTGATAATAGT----GCGAATTAAATGACCATTTT
Pf-msxb-b6.5 line         CGAACACTAGCAAAAATAAATTTTGCCAACAGTATTTGGGGCAAAAAAAGTTACCTTCGC
                          *       *  *     **** ** *  * * ** *    ** **  ** * *** *

Pm-msxb-b6.5 line         A-AGCGTTGTTTTCTATTTGTACAA-TATAATCGAACAACTACAAACGTTTTATTAATAC
Pm-msxb-b6.5 line_tested  A-AGCGTTGTTTTCTATTTGTACAA-TATAATCGAACAACTACAAACGTTTTATTAATAC
Pf-msxb-b6.5 line         AGAGTGTTTGTTTATATATGTCCAAATATATCTATTTATTTGCTGTGGCCCTATCA---C
                          * ** ***  *** *** *** *** ****       *  * *    *   *** *   *

Pm-msxb-b6.5 line         TAACGTAAGTTACATATTGATTTTGCGTAGTTGTTTTATTATTCTTAACGCTTGCTCTGA
Pm-msxb-b6.5 line_tested  TAACGTAAGTTACATATTGATTTTGCGTAGTTGTTTTATTATTCTTAACGCTTGCTCTGA
Pf-msxb-b6.5 line         AAACATAAGT-----ATTGATTTTGCGTAGTTGTTTTATTATTCTTCATGTTTGC-----
                           *** *****     ******************************* * * ****

Pm-msxb-b6.5 line         TTCTTGACACGAGACATTTTGCAGTCATTCATTGGCTCATTATCTGAAGATAATGACTGT
Pm-msxb-b6.5 line_tested  TTCTTGACACGAGACATTTTGCAGTCATTCATTGGCTCATTATCTGAAGATAATGACTGT
Pf-msxb-b6.5 line         -----AGTACGAGACGTCTCGCAGCCATT-ATTGACTCATTATCTTAGGATAACGACTGT
                                  ******* * * **** **** **** ********** * ***** ******

Pm-msxb-b6.5 line         TTGGGTTCATTAATCCAGTTTTCCCTCTAGACTGAGAACCGAATTATAAGTCGATTGCGC
Pm-msxb-b6.5 line_tested  TTGGGTTCATTAATCCAGTTTTCCCTCTAGACTGAGAACCGAATTATAAGTCGATTGCGC
Pf-msxb-b6.5 line         TTGGCTTCGTTAATCCAGTTTTCCCTCCAGACCCAGGACGGAATTATAAGTCGATTGCGC
                          **** *** ****************** ****  ** ** ********************

Pm-msxb-b6.5 line         TAGCCGAACGTGCTTAACGAAACAAAATGGCCGTAAGCCCCATCCCGAATTGACATCCAA
Pm-msxb-b6.5 line_tested  TAGCCGAACGTGCTTAACGAAACAAAATGGCCGTAAGCCCCATCCCGAATTGACATCCAA
Pf-msxb-b6.5 line         TAGCCGAACCCGCTTAACGAAACAAAATGGCCGTAAGCCCCATCCCGAATTGACAACCAG
                          *********  ******************************************** ***

Pm-msxb-b6.5 line         CCAGGGTGCCAGTGCTCGTGGGTTGATTTTAATTGACAGCGTTAATCCCCATCATTGTGG
Pm-msxb-b6.5 line_tested  CCAGGGTGCCAGTGCTCGTGGGTTGATTTTAATTGACAGCGTTAATCCCCATCATTGTGG
Pf-msxb-b6.5 line         CCAGGGTGCCACTGCTCGTCGGGTGATTTTAATTGACAGCGTTAATCCCCATCATTGCGG
                          *********** ******* ** ********************************** **

Pm-msxb-b6.5 line         CAGCTCCGGCAATCCGGACGTCTTGATGAAAAGCGGTACCGCGCAGCGAGTGACCGTCTT
Pm-msxb-b6.5 line_tested  CAGCTCCGGCAATCCGGACGTCTTGATGAAAAGCGGTACCGCGCAGCGAGTGACCGTCTT
Pf-msxb-b6.5 line         CAGCGCGGGCAATCCGGACGTCTTGATGAAAAGCGGTGCCGCGCAGCGAGTGACCGTATT
                          **** * ****************************** ******************* **

Pm-msxb-b6.5 line         TAAGTGTTGCAAACAAGACTTAAACAAACAAGAGAAAGACTCGTGGGGACAAACCCAGCA
Pm-msxb-b6.5 line_tested  TAAGTGTTGCAAACAAGACTTAAACAAACAAGAGAAAGACTCGTGGGGACAAACCCAGCA
Pf-msxb-b6.5 line         TAAGTGTTGCAAACAAGACTTAAACAAACAAGAGAAAGACTCGTAGGAAGAAACCCGGCA
                          ******************************************** ** * ****** ***

Pm-msxb-b6.5 line         GACGAGAGAAAACGACGCGCGCATGGCGACGCACAATGAACGGCGGAGAGTGT
Pm-msxb-b6.5 line_tested  GACGAGAGAAAATGACGCGCGCATGGCGACGCACAATGAACGGCGGGGAGTGT
Pf-msxb-b6.5 line         GACGAGTAAGGACGACGCGCGCGTTGGGACGCACAATGAGCATCGGAGAGTGT
                          ******  *  * ********* * * ************ *  *** ******
```
