# Supplementary material for: An Otx/Nodal Regulatory Signature for Posterior Neural Development in Ascidians
Source: PLoS Genet. 2014 Aug 14;10(8):e1004548. doi: 10.1371/journal.pgen.1004548 (PMC4133040; doi:10.1371/journal.pgen.1004548)
Supplement: Table S1 — Transcriptional activity of enhancers tested in C. intestinalis embryos. (PDF) [file pgen.1004548.s012.pdf]

Table S1 : Analysis of transcriptional activity of constructs electroporated into *Ciona intestinalis* embryos. The numbers of embryos with staining in the b6.5 lineage is determined after X-gal staining.

Gene	Construct	Stage	Number of experiments	(n)	% of stained embryos in the b6.5 lineage
Ci-Msxb	**Ci-msxb-b6.5 line**	early gastrula	2	191	23 %
		late gastrula	2	282	78 %
		neurula	2	256	91 %
		tailbud	2	433	74 %
	**Ci-msxb-OtxUP**	early gastrula	2	300	0 %
		late gastrula	6	1177	34 %
		neurula	3	543	67 %
		tailbud	5	844	70 %
	**Ci-msxb-A**	late gastrula	3	601	60 %
	**Ci-msxb-B**	late gastrula	7	1353	55 %
	**Ci-msxb-B-inv**	late gastrula	3	706	80 %
	**Ci-msxb-D**	late gastrula	7	1449	31 %
	**Ci-msxb-E**	late gastrula	5	966	51 %
	**Ci-msxb-F**	late gastrula	5	1007	12 %
	**Ci-msxb-G**	late gastrula	3	476	57 %
	**Ci-msxb-H**	late gastrula	3	410	55 %
	**Ci-msxb-I**	late gastrula	2	198	0 %
	**Ci-msxb-J**	late gastrula	2	180	0 %
	**Ci-msxb-L**	late gastrula	2	479	3 %
	**Ci-msxb-M**	late gastrula	2	440	0 %
Ci-Delta2	**Ci-delta2-b6.5 line**	early gastrula	2	260	17 %
		late gastrula	4	596	76 %
		neurula	1	98	83 %
		tailbud	4	685	81 %
Pm-Msxb	**Pm-msxb-b6.5 line**	early gastrula	1	98	0 %
		late gastrula	3	589	71 %
		neurula	1	178	84 %
		tailbud	3	557	83 %
