# Supplementary material for: An Otx/Nodal Regulatory Signature for Posterior Neural Development in Ascidians
Source: PLoS Genet. 2014 Aug 14;10(8):e1004548. doi: 10.1371/journal.pgen.1004548 (PMC4133040; doi:10.1371/journal.pgen.1004548)
Supplement: Table S2 — Transcriptional activity of enhancers tested in P. mammillata embryos. (PDF) [file pgen.1004548.s013.pdf]

Table S2 : Analysis of transcriptional activity of constructs electroporated into *Phallusia mammillata* embryos. The numbers of embryos with staining in the b6.5 lineage was determined after X-gal staining.

Gene	Construct	Stage	Number of experiments	(n)	% of stained embryos in the b6.5 lineage
Ci-Msxb	**Ci-msxb-b6.5 line**	neurula	4	1065	40 %
		tailbud	1	225	43 %
Ci-Delta2	**Ci-delta2-b6.5 line**	neurula	4	692	36 %
		tailbud	1	183	7 %
Pm-Msxb	**Pm-msxb-b6.5 line**	neurula	4	628	41 %
		tailbud	1	123	41 %
