# Supplementary material for: An Otx/Nodal Regulatory Signature for Posterior Neural Development in Ascidians
Source: PLoS Genet. 2014 Aug 14;10(8):e1004548. doi: 10.1371/journal.pgen.1004548 (PMC4133040; doi:10.1371/journal.pgen.1004548)
Supplement: Table S3 — Primers and vectors used in transcriptional assays. (PDF) [file pgen.1004548.s014.pdf]

Table S3 : List of primers and vectors used to generate expression clones for transcriptional assay.

Region name	Forward primer	Reverse primer	pENTRY generation method	pDEST
Ci-msxb-b6.5 line	AGCAATCATTTTGAGCTTCCAC	ACTACTCGTCTGAGGGATGC	PCR8/GW/TOPO TA cloning	pSP1.72-RfA-bpFOG-nlsLacZ
Ci-msxb-OtxUP[#]	*ATAAAGTAGGCT*ggagatacgggattaatacaatgaatg	*GAAAAGTTGGGT*ttttctcccgatatttccccattac	BP reaction with pDONR221-P3-P4	pSP72BSSPE-R3-ccdB/cmR-R4-bpFOGB5-B1-nlsLacZ-B2
Ci-delta2-b6.5 line[#]	*ATAAAGTAGGCT*actgtcgcgctttattttag	*GAAAAGTTGGGT*ttgctgatttttttgcgac	BP reaction with pDONR221-P3-P4	pSP72BSSPE-R3-ccdB/cmR-R4-bpFOGB5-B1-nlsLacZ-B2
Pm-msxb-b6.5 line[#]	*ATAAAGTAGGCT*tgttgaaatagtttgaaatcttcctg	*GAAAAGTTGGGT*acactctccgccgttcatt	BP reaction with pDONR221-P3-P4	pSP72BSSPE-R3-ccdB/cmR-R4-bpFOGB5-B1-nlsLacZ-B2

[#] Genomic PCR was performed in two steps. The first step included the primers described in the above table. In the second step, AttB adaptors were included : AttB3-adaptor (5'-GGGGACAAGTTTGTATA*ATAAAGTAGGCT*-3') and AttB4-adaptor-rev (5'-GGGGACCACTTTGTATA*GAAAAGTTGGGT*-3') (Roure et al., 2007). AttB sequences present in each set of primers are in italic. Underlined sequences represent target-specific sequences.
