# Supplementary material for: An Otx/Nodal Regulatory Signature for Posterior Neural Development in Ascidians
Source: PLoS Genet. 2014 Aug 14;10(8):e1004548. doi: 10.1371/journal.pgen.1004548 (PMC4133040; doi:10.1371/journal.pgen.1004548)
Supplement: File S1 — Sequences of msxb-B enhancer and its mutated versions. (PDF) [file pgen.1004548.s015.pdf]

File S1 : Sequences of G-blocks Gene Fragments used in the study. Underlined are AttB1 and AttB2 sequences for recombination. Putative transcription factor binding sites are colored (Otx : red (GATTA) and orange (GHATTA) ; Fox light blue (AACA) and dark blue (AAACA) ; and Smad Binding Element (AGAC)). Introduced mutations are in lower case.

>Msxb-A
GGGGACAAGTTTGTACAAAAAAGCAGGCTGGAGATACGGGATTAATACAATGAATGTTCAATTAAAAACACAAAAGTTTTAATTTGAATTAATG
TCGGCGCTGCAGACGAGCCGAGAGCCTGTCGGATTTAAATTGTAATCCTCCAACTTTGTCTGGCACTGTTGGTGTAGATGCAATCTGAAAATGG
CGGCTGAGCAGGATCGGGTCGCGGCTGGAACTCCGGCGGGCTCGGGACGCTCATAATTCCGCCGGTAATCCCCGTAACGTCGATGAAAGCGAAC
GCGCCGACAAAAGTGACGAAGATTAAGTGTAACAACATGGTTTAAACAAACAGACTGGAGCAGCGGAGACGAGAGAGAGAGGGAACGTAATCGC
CGCGGCCGAAGCGTGACCAAGTCGTTGTATTGTTATAATAGAAAATCGTTTTATTACCCAGCTTTCTTGTACAAAGTGGTCCCC

>Msxb-B
GGGGACAAGTTTGTACAAAAAAGCAGGCTGTCGGCGCTGCAGACGAGCCGAGAGCCTGTCGGATTTAAATTGTAATCCTCCAACTTTGTCTGGC
ACTGTTGGTGTAGATGCAATCTGAAAATGGCGGCTGAGCAGGATCGGGTCGCGGCTGGAACTCCGGCGGGCTCGGGACGCTCATAATTCCGCCG
GTAATCCCCGTAACGTCGATGAAAGCGAACGCGCCGACAAAAGTGACGAAGATTAAGTGTAACAACATGGTTTAAACAAACAGACTGGAGCAGC
GGAGACGAGAGAGAGAGGGAACCCAGCTTTCTTGTACAAAGTGGTCCCC

>Msxb-B-inv
GGGGACAAGTTTGTACAAAAAAGCAGGCTTCCCTCTCTCTCTCGTCTCCGCTGCTCCAGTCTGTTTGTTTAAACCATGTTGTTACACTTAATCT
TCGTCACTTTTGTCGGCGCGTTCGCTTTCATCGACGTTACGGGGATTACCGGCGGAATTATGAGCGTCCCGAGCCCGCCGGAGTTCCAGCCGCG
ACCCGATCCTGCTCAGCCGCCATTTTCAGATTGCATCTACACCAACAGTGCCAGACAAAGTTGGAGGATTACAATTTAAATCCGACAGGCTCTC
GGCTCGTCTGCAGCGCCGACACCCAGCTTTCTTGTACAAAGTGGTCCCC

>Msxb-D
GGGGACAAGTTTGTACAAAAAAGCAGGCTGTCGGCGCTGCAGACGAGCCGAGAGCCTGTCGGATTTAAATTGTAAgCCTCCAACTTTGTCTGGC
ACTGTTGGTGTAGATGCAATCTGAAAATGGCGGCTGAGCAGGATCGGGTCGCGGCTGGAACTCCGGCGGGCTCGGGACGCTCATAATTCCGCCG
GTAAgCCCCGTAACGTCGATGAAAGCGAACGCGCCGACAAAAGTGACGAAGcTTAAGTGTAACAACATGGTTTAAACAAACAGACTGGAGCAGC
GGAGACGAGAGAGAGAGGGAACCCAGCTTTCTTGTACAAAGTGGTCCCC

>Msxb-E
GGGGACAAGTTTGTACAAAAAAGCAGGCTGTCGGCGCTGCAGACGAGCCGAGAGCCTGTCGGATTTAAATTGTAATCCTCCAACTTTGTCTGGC
ACTGTTGGTGTAGATGCAATCTGAAAATGGCGGCTGAGCAGGATCGGGTCGCGGCTGGAACTCCGGCGGGCTCGGGACGCTCATAATTCCGCCG
GTAATCCCCGTAACGTCGATGAAAGCGAACGCGCCGACAAAAGTGACGAAGATTAAGTGTAACAACATGGTTTAAACgAACAGACTGGAGCAGC
GGAGACGAGAGAGAGAGGGAACCCAGCTTTCTTGTACAAAGTGGTCCCC

>Msxb-F
GGGGACAAGTTTGTACAAAAAAGCAGGCTGTCGGCGCTGCAGACGAGCCGAGAGCCTGTCGGATTTAAATTGTAAgCCTCCAACTTTGTCTGGC
ACTGTTGGTGTAGATGCAATCTGAAAATGGCGGCTGAGCAGGATCGGGTCGCGGCTGGAACTCCGGCGGGCTCGGGACGCTCATAATTCCGCCG
GTAAgCCCCGTAACGTCGATGAAAGCGAACGCGCCGACAAAAGTGACGAAGcTTAAGTGTAACAACATGGTTTAAACgAACAGACTGGAGCAGC
GGAGACGAGAGAGAGAGGGAACCCAGCTTTCTTGTACAAAGTGGTCCCC

>Msxb-G
GGGGACAAGTTTGTACAAAAAAGCAGGCTGTCGGCGCTGCAGACGAGCCGAGAGCCTGTCGGATTTAAATTGTAATCCTCCAACTTTGTCTGGC
ACTGcTGGTGTAGATGCAATCTGAAAATGGCGGCTGAGCAGGATCGGGTCGCGGCTGGAACTCCGGCGGGCTCGGGACGCTCATAATTCCGCCG
GTAATCCCCGTAACGTCGATGAAAGCGAACGCGCCGACAAAAGTGACGAAGATTAAGTGTAgCAgCATGGTTTAAgCAAgCAGACTGGAGCAGC
GGAGACGAGAGAGAGAGGGAACCCAGCTTTCTTGTACAAAGTGGTCCCC

>Msxb-H
GGGGACAAGTTTGTACAAAAAAGCAGGCTGTCGGCGCTGCAGACGAGCCGAGAGCCTGTCGGATTTAAATTGTAATCCTCCAACTTTGTCTGGC
ACTGTTGGTGTAGATGCAATCTGAAAATGGCGGCTGAGCAGGATCGGGTCGCGGCTGGAACTCCGGCGGGCTCGGGACGCTCATAATTCCGCCG
GTAATCCCCGTAACGTCGATGAAAGCGAACGCGCCGACAAAAGTGACGAAGATTAAGTGTAACAACATGGTTTAAgCAAgCAGACTGGAGCAGC
GGAGACGAGAGAGAGAGGGAACCCAGCTTTCTTGTACAAAGTGGTCCCC

>Msxb-I
GGGGACAAGTTTGTACAAAAAAGCAGGCTGTCGGCGCTGCAGACGAGCCGAGAGCCTGTCGGATTTAAATTGTAcgCCTCCAACTTTGTCTGGC
ACTGTTGGTGTAGATGCAATCTGAAAATGGCGGCTGAGCAGGATCGGGTCGCGGCTGGAACTCCGGCGGGCTCGGGACGCTCATAcgTCCGCCG
GTAcgCCCCGTAACGTCGATGAAAGCGAACGCGCCGACAAAAGTGACGAAGcgTAAGTGTAACAACATGGTTTAAACAAACAGACTGGAGCAGC
GGAGACGAGAGAGAGAGGGAACCCAGCTTTCTTGTACAAAGTGGTCCCC

>Msxb-J
GGGGACAAGTTTGTACAAAAAAGCAGGCTGTCGGCGCTGCAGACGAGCCGAGAGCCTGTCGGATTTAAATTGTAcgCCTCCAACTTTGTCTGGC
ACTGcTGGTGTAGATGCAATCTGAAAATGGCGGCTGAGCAGGATCGGGTCGCGGCTGGAACTCCGGCGGGCTCGGGACGCTCATAcgTCCGCCG
GTAcgCCCCGTAACGTCGATGAAAGCGAACGCGCCGACAAAAGTGACGAAGcgTAAGTGTAgCAgCATGGTTTAAgCAAgCAGACTGGAGCAGC
GGAGACGAGAGAGAGAGGGAACCCAGCTTTCTTGTACAAAGTGGTCCCC

>Msxb-L
GGGGACAAGTTTGTACAAAAAAGCAGGCTGTCGGCGCTGCctACGAGCCGAGAGCCTGTCGGATTTAAATTGTAATCCTCCAACTTTGTagGGC
ACTGTTGGTGTAGATGCAATCTGAAAATGGCGGCTGAGCAGGATCGGGTCGCGGCTGGAACTCCGGCGGGCTCGGGACGCTCATAATTCCGCCG
GTAATCCCCGTAACGTCGATGAAAGCGAACGCGCCGACAAAAGTGACGAAGATTAAGTGTAACAACATGGTTTAAACAAACctACTGGAGCAGC
GGctACGAGAGAGAGAGGGAACCCAGCTTTCTTGTACAAAGTGGTCCCC

>Msxb-M
GGGGACAAGTTTGTACAAAAAAGCAGGCTGTCGGCGCTGCctACGAGCCGAGAGCCTGTCGGATTTAAATTGTAAGCCTCCAACTTTGTagGGC
ACTGTTGGTGTAGATGCAATCTGAAAATGGCGGCTGAGCAGGATCGGGTCGCGGCTGGAACTCCGGCGGGCTCGGGACGCTCATAATTCCGCCG
GTAAGCCCCGTAACGTCGATGAAAGCGAACGCGCCGACAAAAGTGACGAAGCTTAAGTGTAACAACATGGTTTAAACAAACctACTGGAGCAGC
GGctACGAGAGAGAGAGGGAACCCAGCTTTCTTGTACAAAGTGGTCCCC
